# Derivatisable Cyanobactin Analogues: A Semisynthetic Approach

**DOI:** 10.1002/cbic.201500494

**Published:** 2015-11-10

**Authors:** Emilia Oueis, Catherine Adamson, Greg Mann, Hannes Ludewig, Philip Redpath, Marie Migaud, Nicholas J. Westwood, James H. Naismith

**Affiliations:** ^1^ Biomedical Sciences Research ComplexUniversity of St. AndrewsNorth HaughSt. AndrewsKY16 9STUK; ^2^ John King Medicinal Chemistry LaboratorySchool of PharmacyQueen's University97 Lisburn RoadBelfastBT9 7BLUK; ^3^ State Key Laboratory of BiotherapySichuan UniversityChengduSichuanChina

**Keywords:** click chemistry, cyclic peptides, enzymatic reactions, macrocyclisation, patellamides

## Abstract

Many natural cyclic peptides have potent and potentially useful biological activities. Their use as therapeutic starting points is often limited by the quantities available, the lack of known biological targets and the practical limits on diversification to fine‐tune their properties. We report the use of enzymes from the cyanobactin family to heterocyclise and macrocyclise chemically synthesised substrates so as to allow larger‐scale syntheses and better control over derivatisation. We have made cyclic peptides containing orthogonal reactive groups, azide or dehydroalanine, that allow chemical diversification, including the use of fluorescent labels that can help in target identification. We show that the enzymes are compatible and efficient with such unnatural substrates. The combination of chemical synthesis and enzymatic transformation could help renew interest in investigating natural cyclic peptides with biological activity, as well as their unnatural analogues, as therapeutics.

Ribosomally synthesised and post‐translationally modified peptides (RiPPs) make up a wide group of natural compounds with various biological activities.^1^ The biosynthetic pathway of RiPPs involves the action of many tailoring enzymes on a specific precursor peptide to yield the highly modified final product. Cyanobactins are a large family of RiPPs that includes patellamides, ulithiacyclamides, and trunkamides (Scheme 1) and that are produced by a diverse selection of cyanobacteria.^2^ Amongst the best‐studied cyanobactins are the patellamides: cyclic octapeptides produced by *Prochloron didemni*, the cyanobacterial symbiont of *Lissoclinum patella*. The biosynthetic pathway of patellamides consists of a seven‐gene cluster (*patA–G*; Figure 1) that encodes the precursor peptide (PatE) as well as the altering enzymes. Common modifications of patellamides include heterocyclisation, oxidation, epimerisation and macrocyclisation.^3^

**Scheme 1:**
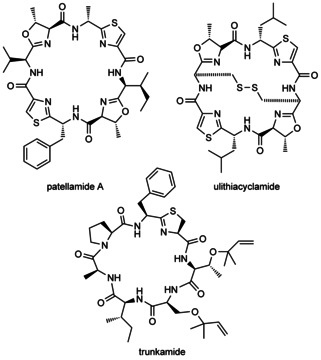
Structures of cyanobactin metabolites.

**Figure 1:**
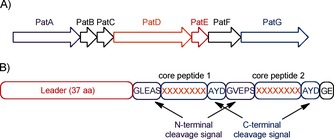
A) The *pat* gene cluster codes for PatA, which cleaves N‐terminal to core peptide, PatB and PatC (unknown function), PatD, which heterocyclases cysteine (serine, threonine) residues in the core peptide, PatE (precursor peptide), PatF (inactive prenylase^7^) and PatG, which cleaves and macrocyclises to the core peptide and oxidises thiazolines. B) PatE precursor peptide with its key regions highlighted.

Cyanobactins, including patellamides, have diverse and valuable biological activities.2b Patellamides B, C and D have shown a reversal of the multidrug resistance seen for vinblastine, colchicine and adriamycin treatment in the CEM/VLB_100_ human leukemic cell line,^4^ and patellamide D is cytotoxic towards fibroblast (MRC5V1) and bladder calcinoma (T24) tumour cell lines.^5^ However, the development of these compounds requires large‐scale synthesis in order to identify their exact biological targets and ascertain structure–activity relationships to fine‐tune their properties. The chemical synthesis of such compounds is challenging, even more so (>16 synthetic steps) for those containing thiazoline and/or oxazoline moieties.^6^

Biochemical studies have shown that PatG_mac_ (macrocyclisation domain of PatG) tolerates significant diversity in the amino acids in the core peptide,^8^ as long as the core peptide ends with a five‐membered heterocyclic ring (either the naturally occurring thiazoline/oxazoline motif or *cis‐*proline) and is flanked by a C‐terminal macrocyclisation signature AYD.^9^ More recently, an engineered heterocyclase that can completely process peptide substrates lacking the leader peptide has been reported.^10^ Taking advantage of this efficient biosynthetic machinery, we show that pairing chemical and enzymatic syntheses is efficient for the generation of unnatural patellamide‐like cyclic peptides. We report herein the macrocyclisation of synthetic peptides that contain unnatural amino acids by using PatG_mac_ as well as the selective derivatisation of the subsequent cyclic peptides. We also report the synthesis of a patellamide‐like cyclic peptide containing both a heterocycle and an unnatural amino acid in a one‐pot procedure.

The introduction of bio‐orthogonal or specific reacting groups on the side chains of linear/cyclic peptide residues would be highly desirable, as they would allow regiospecific and facile derivatisation. This approach has been previously used to study binding and/or activity,^11^ to link a fluorescent probe in order to investigate biological processes or pharmacokinetic behaviour,^12^ and to connect other building blocks for activity enhancement or drug delivery,^13^ among others. Although orthogonal reacting groups have been previously introduced in vivo on precursor peptides of RiPPs through stop‐codon suppression (SCS) and supplementation‐based incorporation (SPI),^14^ these strategies lack the control for better selectivity, specificity and flexibility that chemical synthesis permits. Three patellamide‐like cyclic peptides were made with either an azidoalanine A(N_3_) or a dehydroalanine (Dha) reactive group. The azide moiety is a well known bio‐orthogonal group that reacts with alkynes both ex and in vivo.^15^ Although not fully bio‐orthogonal, Dha is extensively used for bioconjugation purposes.^16^ The two groups, A(N_3_) and Dha, were introduced at different positions (4 and 2, respectively) in their respective core peptides to explore whether their incorporation presented any challenges for PatG_mac_ processing. For simplicity, we made the first two compounds from peptides that lacked any cysteine residues, and therefore heterocycles. Having established a suitable approach, we advanced to adding the azide to a cysteine‐containing peptide. We were able to enzymatically heterocyclise the cysteine to a thiazoline within the sequence and then macrocyclise the resulting product in a one‐pot process, to make a genuine patellamide analogue.

The synthetic precursor peptides **1** (ITAA(N_3_)ITAPAYD/G) and **3** (VDhaAGIGFPAYDG; Schemes S1–S2; Figures S2–S5 in the Supporting Information) were incubated in the presence of PatG_mac_. The corresponding cyclic peptides cyclo(‐ITAA(N_3_)ITAP‐) (**2**) and cyclo(‐VDhaAGIGFP‐) (**4**) were obtained in 45 and 63 % purified yield, respectively (Scheme 2). To prove their macrocyclic structure, peptides **2** and **4** were extensively analysed by NMR spectroscopy and MS^2^ (Supporting Information). Furthermore, the Dha group was found to be highly stable under the macrocyclisation reaction conditions (Figures S31–S32).

**Scheme 2:**
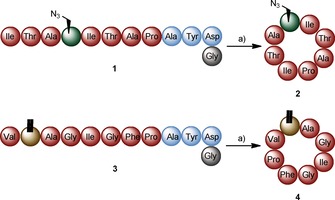
Macrocyclisation of the unnatural amino acid‐containing precursor peptides **1** and **3** with PatGmac affords cyclic peptides **2** (45 % yield) and **4** (63 % yield). a) PatG_mac_, 37 °C, pH 8.1

Purified cyclic peptide **2** was then reacted with cyclooctyne **5** in a copper‐free, strain‐promoted azide–alkyne cycloaddition (Scheme 3).^17^ The product, **6**, was obtained in 95 % yield. We used the same procedure to treat **2** with cyclooctyne **7** (DBCO‐Cy5), which contains the fluorescent cyanine tag (Cy5; Scheme 2), and the cyclic peptide–Cy5 conjugate **8** was obtained in 30 % yield.

**Scheme 3:**
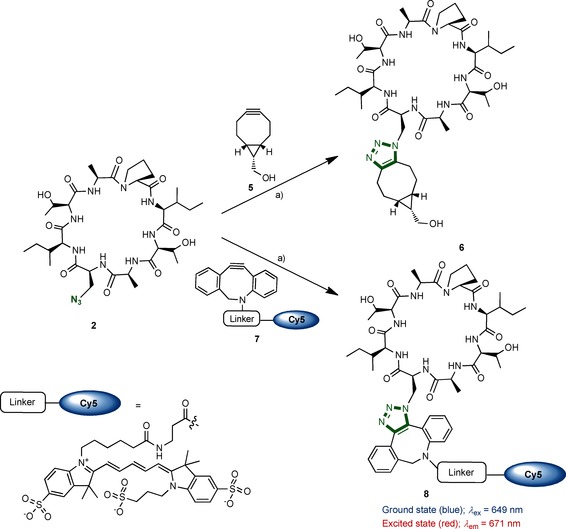
Copper‐free azide–alkyne cycloaddition of **2** with strained cyclooctynes affords conjugates **6** (95 % yield) and **8** (30 % yield). a) CH_3_CN, H_2_O.

The measured maximum absorbance (*λ*=649 nm) and emission (*λ*=671 nm) properties of conjugate **8** were in good agreement with those of the parent Cy5 molecule (Figure S33). As these types of compounds could potentially be used for target identification by fluorescence microscopy, conjugate **8** (dark blue colour) was tested to ensure there was no unexpected behaviour (such as quenching or precipitation) in cells. When incubated with permeabilised HeLa cells, a diffuse staining pattern of **8** (red colour) throughout the cytoplasm and nucleus was visualised by fluorescent microscopy (Figure 2); this showed that the molecule behaves as expected in biological buffers.

**Figure 2:**
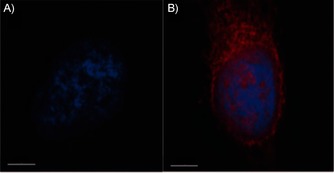
A) DMSO control nucleus stained with Dapi (blue); B) Cyclic peptide **8** visualised in fixed permeabilised HeLa cells (red; pink when overlapped with nucleus). Scale bars: 5 μm.

Cyclic peptide **4** underwent a thio‐Michael addition with the cysteine‐containing glutathione peptide **9** (Scheme 4) with an excess of triethylamine in water and methanol.^18^ The corresponding compound **10** was obtained in 43 % yield. Following the successful addition of glutathione, we investigated whether the reaction could be carried out directly after the macrocyclisation reaction as a one‐pot process. Once peptide **3** has been fully macrocyclised, 100 equivalents of mercaptoethanol were directly added into the reaction mixture, and this was left at 37 °C overnight. The reaction was judged to be complete by MS, and the final compound **11** was obtained in 60 % yield. The final product purifies as two separable peaks, which we attribute to different diastereoisomers (Figure S34).

**Scheme 4:**
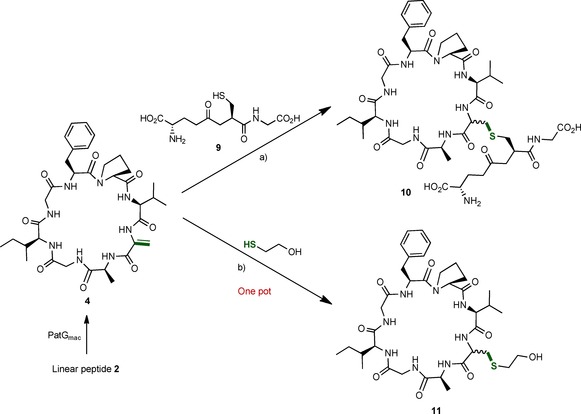
Thio‐Michael addition of thiol derivatives to cyclic peptide **4**. a) Et_3_N, H_2_O/MeOH (43 %); b) Enzymatic reaction, bicine/NaCl buffer (pH 8.1), 37 °C (60 %).

As PatG_mac_ processes substrates with unnatural amino acids at similar rates to other sequences,^9^ we next tested the feasibility of introducing heterocycles into such unnatural substrates. The proline residue in peptide **1** was replaced with a cysteine (peptide **12**) that could be enzymatically heterocyclised. Like PatG_mac_, the heterocyclase enzymes of the cyanobactin pathways (known as the D enzymes)3a, 19 have been shown to be tolerant of a wide range of sequences within the core peptide.

We incubated peptide **12** overnight with the engineered heterocyclase LynDfusion (from the aestuaramide pathway (*Lyngbya* sp.)) in the presence of ATP and MgCl_2_. The fully heterocyclysed product **13** was detected by MS but not isolated (Scheme 5; Figures S35–S36). Subsequent addition of PatG_mac_ to the reaction mixture afforded the patellamide‐like analogue **14** cyclo(‐ITAA(N_3_)ITA^het^C‐) in 58 % yield.^20^

**Scheme 5:**
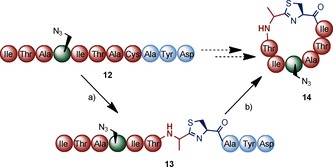
One‐pot heterocyclisation with LynDfusion and macrocyclisation with PatG_mac_ for the synthesis of the unnatural patellamide **14**. a) LynDfusion, 27 °C, pH 9; b) PatG_mac_, 27 °C, pH 8.1.

Milligram quantities of cyanobactin derivatives with fluorescent components will greatly facilitate the target identification of many of these natural biologically active products.2b Target identification will not only provide a basis for redesign of the natural product but could also disclose new opportunities for therapy. The expense and complexity of these labels means in practical terms that they are better introduced late in the synthesis. In the case of macrocyclic peptides, this means ideally after the macrocycle is made. Introducing chemical diversity to probe or fine tune the pharmacokinetic and biological properties of natural products is likewise most desirable when performed as a final step on a common scaffold.

We have demonstrated that both the heterocyclases and macrocyclases from the patellamide (and a related) pathway can be used in vitro with entirely synthetic substrates that contain such chemically reactive unnatural amino acids. Moreover, we have shown that the resulting macrocycles can be derivatised with high efficiency. The ability to combine the diversity of chemical synthesis with the exquisite catalysis of enzymes is well known and recognised to be powerful in developing natural products into therapeutics.^21^ This approach can be extended to peptidic macrocycles and might likewise enable their further development.

## Supporting information

As a service to our authors and readers, this journal provides supporting information supplied by the authors. Such materials are peer reviewed and may be re‐organized for online delivery, but are not copy‐edited or typeset. Technical support issues arising from supporting information (other than missing files) should be addressed to the authors.

Supplementary
