# Supplementary material for: Derivatisable Cyanobactin Analogues: A Semisynthetic Approach
Source: Chembiochem. 2015 Nov 10;16(18):2646–50. doi: 10.1002/cbic.201500494 (PMC4736454; doi:10.1002/cbic.201500494)
Supplement: Supplementary file 1 — Supplementary [file CBIC-16-2646-s001.pdf]

CHEMBIOCHEM

Supporting Information

Derivatisable Cyanobactin Analogues: A Semisynthetic Approach

Emilia Oueis,[a] Catherine Adamson,[a] Greg Mann,[a] Hannes Ludewig,[a] Philip Redpath,[b] Marie Migaud,[b] Nicholas J. Westwood,*[a] and James H. Naismith*[a, c]

cbic_201500494_sm_miscellaneous_information.pdf

Table of Contents

I. GENERAL INFORMATION AND MATERIALS S2

II. GENERAL PROCEDURES S4

III. SYNTHETIC PROCEDURES AND ANALYSIS DATA OF CYCLIC PEPTIDES 2, 4, AND 14 .... S11

IV. SYNTHETIC PROCEDURES OF CYCLIC PEPTIDE DERIVATIVES 6, 8, 10, AND 11 .... S28

V. ADDITIONAL FIGURES S29

VI. HPLC TRACES OF COMPOUNDS 2, 4, 6, 8, 10, 11, AND 14 .... S33

VII. REFERENCES S36

I. General information and materials

The HPLC grade acetonitrile (MeCN) and methanol (MeOH) were purchased from VWR. Aqueous buffers and aqueous mobile-phases for HPLC were prepared using water purified with an Elga® Purelab® Milli-Q water purification system (purified to 18.2 MΩ.cm). Other chemicals and reagents were purchased from Sigma and used without any further purification.

NMR spectra (^{1}H, 2D) were recorded on either a Bruker Ultrashield 500 spectrometer {δH (500 MHz), δC (126 MHz)} or a Bruker Ascend 700 spectrometer {δH (700 MHz), δC (175 MHz)} at ambient temperature in the deuterated solvent stated. Chemical shifts are expressed in parts per million (ppm) from DMSO-d6 (δH = 2.50).[1] Multiplicities are described as s (singlet), d (doublet), dd (doublet of doublets), t (triplet), m (multiplet), br (broad). Coupling constants *J* are quoted in Hertz (Hz) to the nearest 0.1 Hz. Signals of protons and carbons were assigned, as far as possible, by using the following two dimensional NMR spectroscopy techniques: [^{1}H, ^{1}H] COSY (Correlation Spectroscopy), [^{1}H, ^{1}H] TOCSY (Total Correlation Spectroscopy), [^{1}H, ^{1}H] ROESY (Rotating-frame NOE Spectroscopy), [^{1}H, ^{13}C] HSQC (Heteronuclear Single Quantum Coherence) and long range [^{1}H, ^{13}C] HMBC (Heteronuclear Multiple Bond Connectivity). EXSY (Exchange Spectroscopy) experiment was used to identify equilibrium chemical exchange when double peaks were observed.

Low-resolution mass spectra were obtained with an Agilent 6130 single quad apparatus equipped with an electrospray ionization source. High-resolution mass spectra (HRMS) were obtained with a Thermo Exactive Orbitrap mass spectrometer.

Infrared (IR) spectra were recorded on a Shimadzu IRAffinity-1 Fourier transform IR spectrophotometer using Pike MIRacle ATR accessory. Analysis was carried out using Shimadzu IRsolution v1.50 and only characteristic peaks are reported in wavenumbers (cm^{-1}). Melting points were recorded in open capillaries using an Electrothermal 9100 melting point apparatus. Values are quoted to the nearest 1 °C and are uncorrected; *dec.* refers to decomposition.

Organic reactions were monitored by analytical LC-MS. Reactions performed in the enzymatic media were monitored using MALDI-MS acquired using a 4800 MALDI TOF/TOF Analyser (ABSciex, Foster City, CA) equipped with a Nd:YAG 355 nm

laser and calibrated using a mixture of peptides. The spot was analysed in positive MS mode between 800 and 4000 m/z, by averaging 1000 laser spots. The samples, diluted in water to reduce the buffer concentration, (0.5 mL) were applied to the MALDI target along with alpha-cyano-4-hydroxycinnamic acid matrix (0.5 mL, 10 mg/mL in 50:50 acetonitrile:0.1% TFA) and allowed to dry. MSMS data were acquired using a TripleTOF 5600+. The sample was subjected to chromatography on an Acclaim PepMap 100 C18 trap and an Acclaim PepMap RSLC C18 column (ThermoFisher Scientific), using a nano-LC Ultra 2D plus loading pump and nano-LC as-2 autosampler (Eksigent). The sample was injected at neutral pH to avoid acid catalyzed ring opening. The trap was washed with 2% acetonitrile, 0.05% trifluoroacetic acid, and the patellamide was then eluted with a gradient of increasing acetonitrile, containing 0.1 % formic acid (15-40% acetonitrile in 5 min, 40-95% in a further 1 min, followed by 95% acetonitrile to clean the column, before re-equilibration to 15% acetonitrile). The eluent was sprayed into a TripleTOF 5600+ electrospray tandem mass spectrometer (Sciex) operating with standard nanospray conditions, and analyzed in Product Ion Scan mode isolating the m/z of interest. The collision energy was adjusted to give optimal fragmentation. The MSMS fragmentation pattern was interrogated for diagnostic peaks.

Analytical RP-HPLC was performed on an Agilent infinity 1260 series equipped with a VWD detector and a single quadrupole MS using a Macherey-Nagel Nucleodur C18 column (10 μm x 4.6 x 250 mm). Several chromatographic systems were used; System A1: 0.5 mL/min flow rate with MeCN and 0.1 % aqueous TFA [95% TFA (5 min), linear gradient from 5 to 95% of MeCN (35 min), 95% MeCN (40 min)] and UV detection at 220 nm (unless otherwise stated). System A2: 1 mL/min flow rate with MeCN and 5 mM aqueous ammonium carbonate (AmC) [95% AmC (5 min), linear gradient from 5 to 95% of MeCN (35 min), 95% MeCN (40 min)] and UV detection at 220 nm. System A3: 1 mL/min flow rate with MeCN and 0.1 % aqueous TFA [95% TFA (5 min), linear gradient from 5 to 50% of MeCN (50 min), linear gradient from 50 to 95% MeCN (60 min), 95% MeCN (65 min)] and UV detection at 600 nm.

Semi-preparative RP-HPLC was performed on an Agilent infinity 1260 series equipped with a VWD detector (and a single quadrupole MS) using a Macherey-Nagel Nucleodur C18 column (10 μm x 10 x 250 mm) or a Phenomenex Luna C18

column (5 μm x 10 x 250 mm). Several chromatographic systems were used; System P1: 4 mL/min flow rate with MeCN and 0.1 % aqueous TFA [95% TFA (5 min), linear gradient from 5 to 95% of MeCN (35 min), 95% MeCN (40 min)] and UV detection at 220 nm. System P2: 4 mL/min flow rate with MeCN and 5 mM aqueous ammonium carbonate (AmC) [95% AmC (5 min), linear gradient from 5 to 95% of MeCN (35 min), 95% MeCN (40 min)] and UV detection at 220 nm. System P3: 4 mL/min flow rate with MeCN and 0.1 % aqueous TFA [95% TFA (5 min), linear gradient from 5 to 30% of MeCN (30 min), then a slower linear gradient from 30 to 32.5% of MeCN (55 min), 95% MeCN (60 min)] and UV detection at 220 nm or 600 nm. System P4: 4 mL/min flow rate with MeCN and 0.1 % aqueous TFA [95% TFA (5 min), linear gradient from 5 to 50% of MeCN (50 min), 95% MeCN (60 min)] and UV detection at 220 nm.

II. General procedures

***Pat*G$_{mac}$ cloning, expression and purification:**

The *Pat*G$_{mac}$ enzyme was cloned from genomic DNA *(Prochlon sp.*) into the pHISTEV vector, expressed in *Escherichia coli* BL21 (DE3) cells grown on autoinduction medium, and purified as previously described by Koehnke *et al.*.[2] However, subsequent to the Nickel column eluting with 250 mM imidazole, the remaining purification steps were replaced with dialysis in a bicine buffered solution [20 mM Bicine, 150 mM NaCl, pH = 8.1] to remove the imidazole and the reducing agent.

***Lyn*Dfusion cloning, expression and purification:**

The *Lyn*Dfusion was cloned, expressed in *Escherichia coli* BL21 (DE3) cells grown on autoinduction medium, and purified as previously described by Koehnke *et al.*.[3]

Cell culture and microscopy

HeLa cells (ATCC) were maintained in Dulbecco Modified Eagle Medium supplemented with 10% (v/v) fetal bovine serum (FBS) (DMEM-10). HeLa cells

were seeded onto coverslips at ~50% confluence and were fixed with 5% formaldehyde in phosphate buffered saline (PBS) for 20 minutes at room temperature. After being washed three times with PBS, cells were permeabilized with PBS containing 0.1% Triton-X-100 for 2 minutes at room temperature, followed by washing as above. Cells were then incubated with the test peptide conjugate **8** at a concentration of 0.002 mM in DMEM-10 (initial concentration of 1 mM in DMSO diluted to 1:500 in buffer). Incubation was performed for 6 hours at 37°C followed by overnight at 4°C. Following the incubation period, cells were washed once with PBS, stained with DAPI (4',6-diamidino-2-phenylindole) and mounted with Prolong Gold antifade reagent (molecular probes). Cells were subsequently visualized with a DeltaVision Elite microscope.

Procedure A: Pat Gmac macrocylisation reaction

The reactions were conducted in 20 mM bicine buffer, 500 mM NaCl, and 5% DMSO solution, pH = 8.1 and incubated at 37 °C on a shaking platform until full consumption of the starting peptide (MALDI monitoring). The reaction set-ups were prepared in the following order; final concentrations:

1- A solution of the linear peptide in DMSO (between 10 and 50 mM); 100 µM
2- DMSO; 5%
3- 20 mM Bicine, 150 mM NaCl, pH=8.1 buffer
4- 5 mM NaCl; 500 mM
5- *Pat*G_{mac} enzyme; 60 µM

The reaction mixture was then extracted 3 times with *n*-buthanol (BuOH). BuOH (1/1, v/v) was added to the aqueous reaction, vigorously mixed, and then centrifuged for 10 min at high speed to help separate the two phases. The combined BuOH fractions evaporated under reduced pressure to dryness. The crude was solubilized in a minimum volume of H_2O/MeCN or H_2O/MeOH for HPLC purification.

Procedure B: Click reaction

The pure azide-containing cyclic peptide (1 eq) and the cyclooctyne (1 eq) derivative were stirred overnight in MeCN and H_2O (4:1, v/v). HPLC monitoring verified the completion of the reaction. The crude mixture was directly purified by HPLC.

Procedure C: Thio-Michael addition

The pure dehydroalanine-containing peptide (1 eq) and glutathione (12 eq) were dissolved in water/MeOH (3:1) then triethylamine (12 eq) was added. The reaction mixture was bubbled with dry argon and stirred overnight at rt under inert atmosphere with light protection. HPLC monitoring verified the completion of the reaction. The crude mixture was directly purified by HPLC.

Procedure D: *In situ* thio-Michael addition

After completion of the macrocyclisation reaction (procedure A) and before extraction, 100 eq. of thioethanol were added to the reaction mixture and left at 37 °C overnight. MALDI monitoring confirmed full conversion of the starting material. The final compound was then extracted with BuOH as explained in procedure A.

Procedure E: One pot heterocyclization/macrocyclization

The heterocyclization reaction was first conducted in 20 mM bicine buffer and 150 mM NaCl solution, pH = 9.0 and incubated at 27 °C in the presence of ATP and Magnesium Chloride ($MgCl_2$) (without shaking) overnight (MALDI monitoring). The reaction set-ups were prepared in the following order; final concentrations:

1- A solution of the linear peptide in DMSO; 110 µM
2- 20 mM Bicine, 150 mM NaCl, pH=9.0 buffer
3- 100 mM ATP; 5 mM
4- 1 M $MgCl_2$; 5 mM
5- *Lyn*Dfusion enzyme; 5 µM

Then, the salt concentration was increased to 500 mM, DMSO was added (to help with the solubilization of products), and *Pat*G_{mac} enzyme was added to the reaction mixture. Below are the final concentrations for the macrocyclization reaction:

1- Peptide; 90 µM
2- DMSO; 5%
3- 5 mM NaCl; 500 mM
4- *Pat*G_{mac} enzyme; 50 µM

Solid-phase peptide synthesis

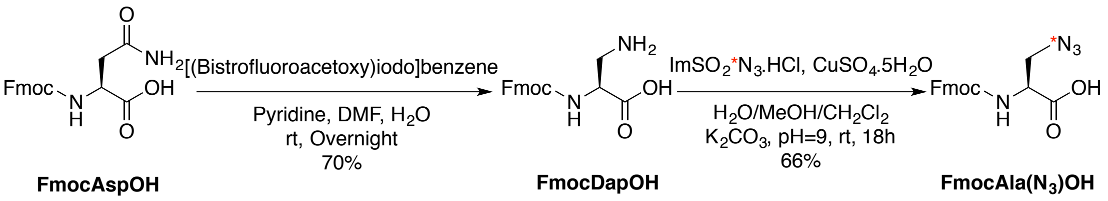

Scheme S1. Synthesis of FmocAla(N_3)OH in two steps from FmocAspOH.

The Fmoc-protected non-natural amino acid L-azidoalanine was synthesized from the Fmoc-protected L-asparagine following the procedure previously described by Spring *et col.*.[4] The $^{15}N_1$-labeled analogue (*) was also synthesized to afford 50% labeling on the final azide. Azidoalanine was later used in the solid phase synthesis of the precursor peptide using the Fmoc strategy.

NMR and MS data are perfectly identical to those described in the literature.[4]

FmocAla(N₃)OH: HRMS (ESI+): Calc. for $C_{18}H_{16}N_4O_4$ $[M+Na]^+$: 375.1064, found: 375.1064 (90%); Calc. for $C_{18}H_{16}N_3{}^{15}NO_4$ $[M+Na]^+$: 376.1034, found: 376.1035 (100%); $HPLC_{254\ nm}$ t_R = 23.22 (purity = 99%; System A1).

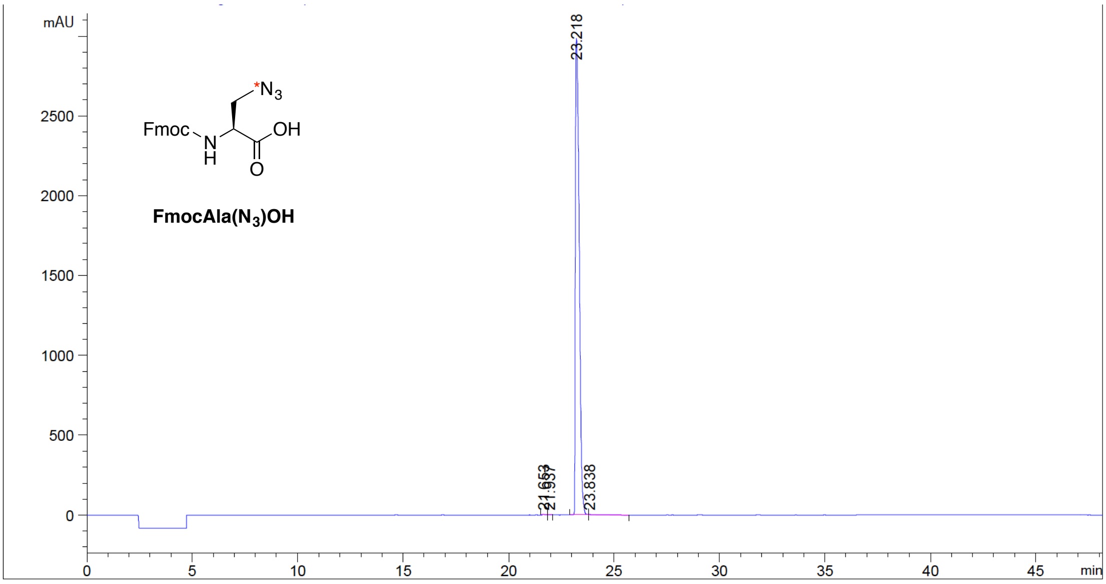

Figure S1. HPLC trace of FmocAla(N_3)OH at 254 nm.

The dehydroalanine (Dha) non-natural amino acid[5] was generated as a final step (after the peptide synthesis) by an elimination reaction of Cys(Me).[6]

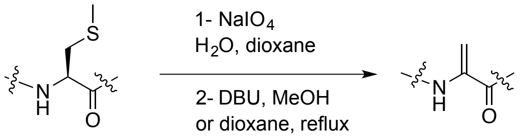

Scheme S2. Synthesis of Dha *via* oxidation and subsequent elimination of Cys(Me).

Starting peptides **1**, **3**, and **12** were synthesized on solid phase using the standard Fmoc strategy on a Rink Amide resin or purchased from Peptide Protein Research Ltd. The final purities of the starting peptides were at least more than 85% as determined by HPLC analysis at 216 nm.

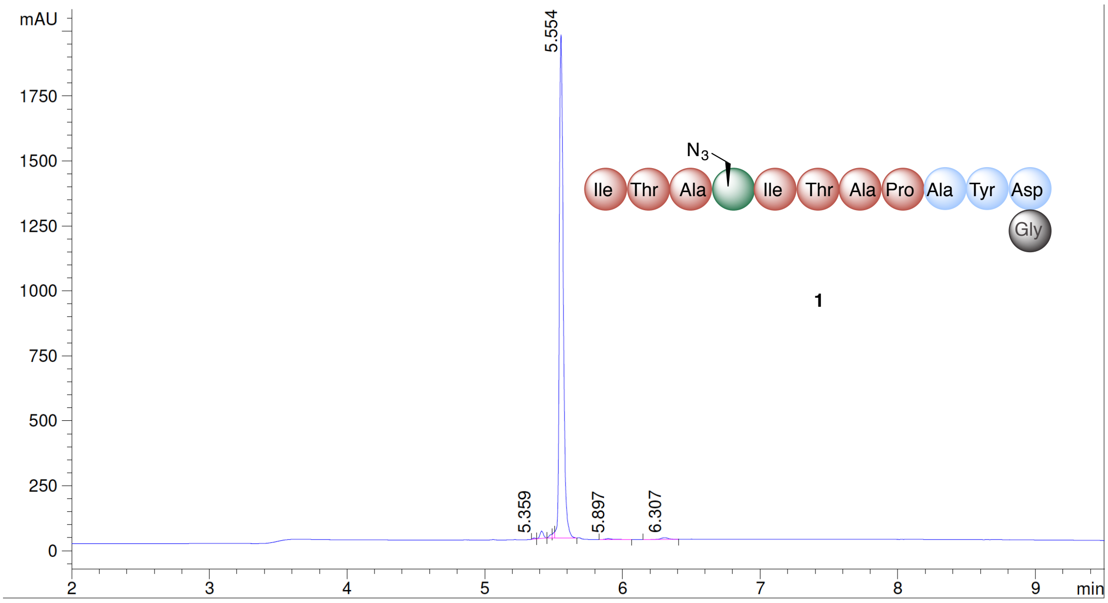

Figure S2: HPLC trace of peptide **1** carried out using a 130Å 4.6 x 50mm column, 10%-90% MeCN gradient (0.1% TFA; 1.5 mL/min), in 8 minutes at 80 °C.

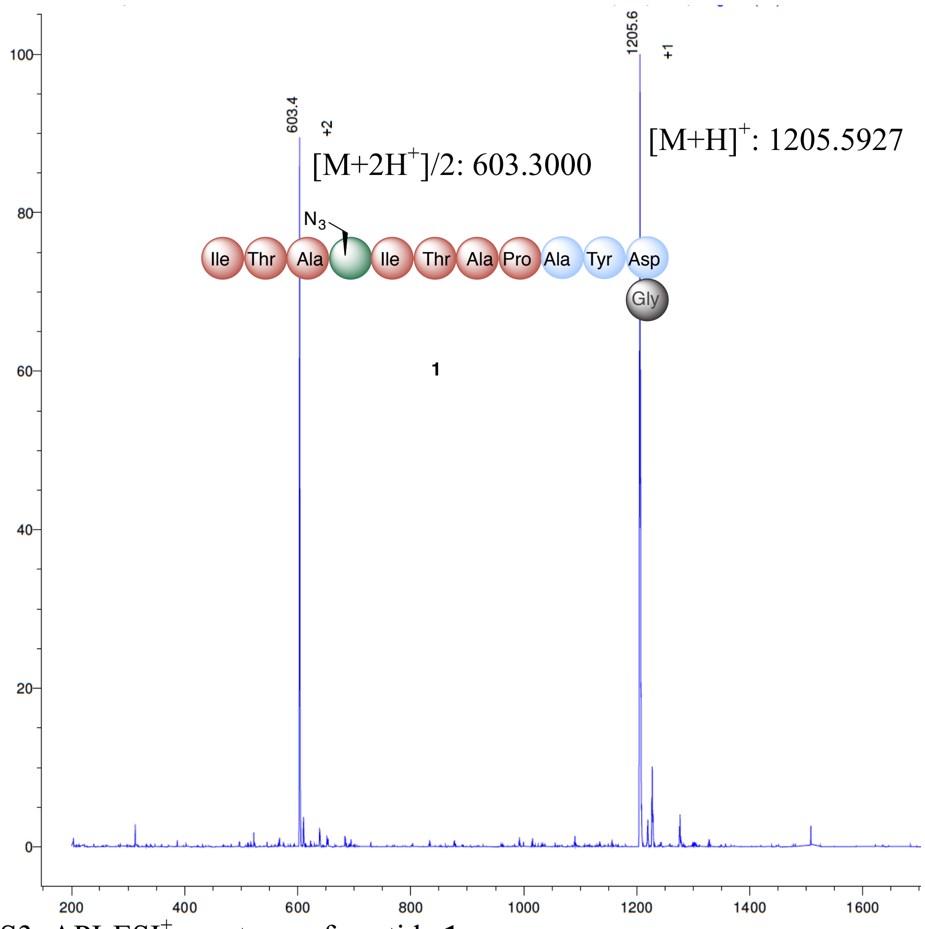

Figure S3: API-ESI$^+$ spectrum of peptide **1**.

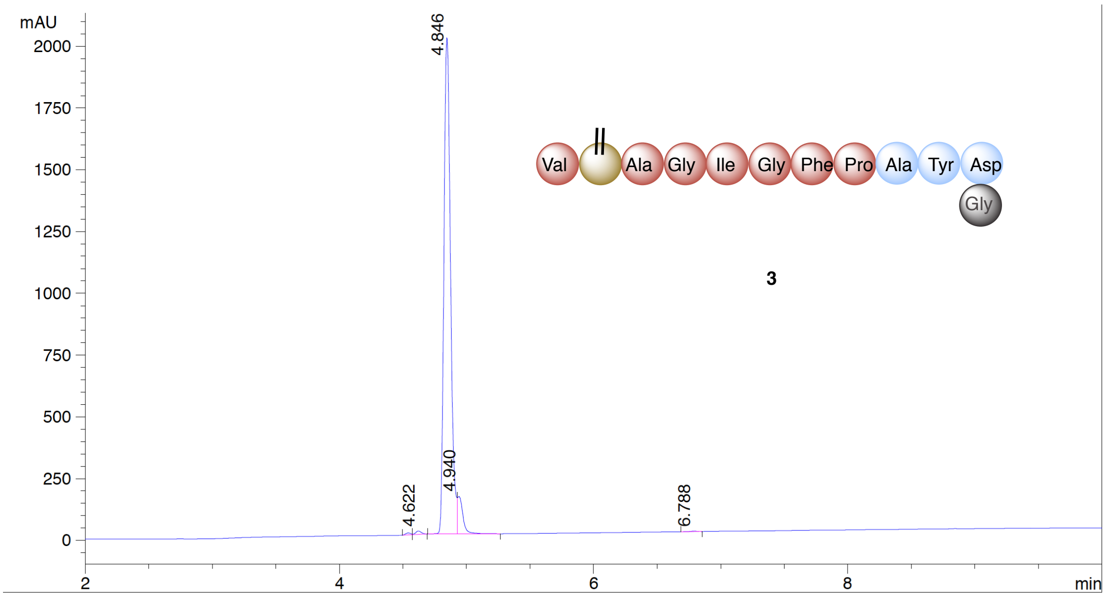

Figure S4: HPLC trace of peptide **3** carried out using a 130Å 4.6 x 50mm column, 10%-90% MeCN gradient (0.5% ammonium acetate; 1.5 mL/min), in 8 minutes at rt.

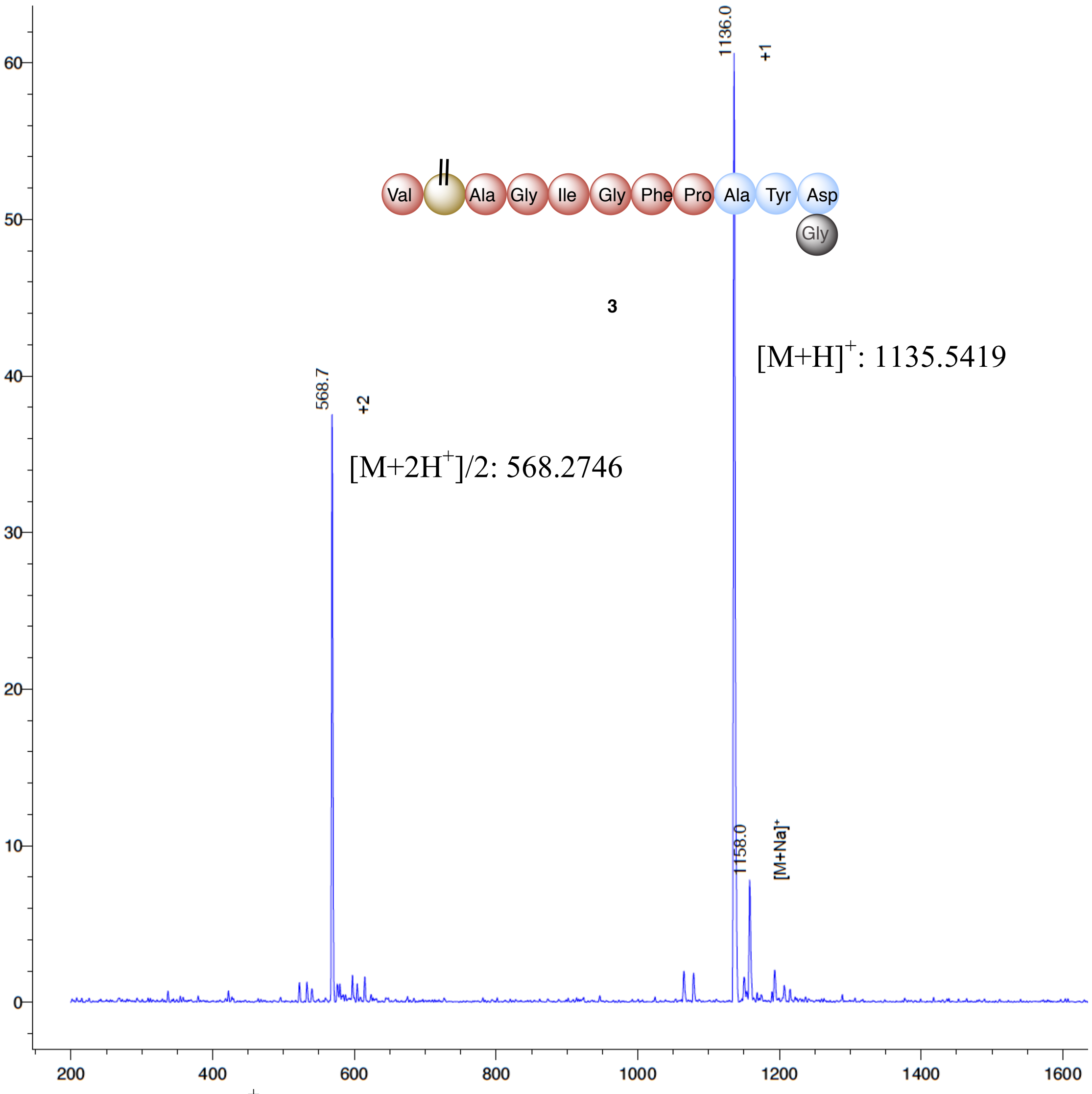

Figure S5: API-ESI$^+$ spectrum of peptide **3**.

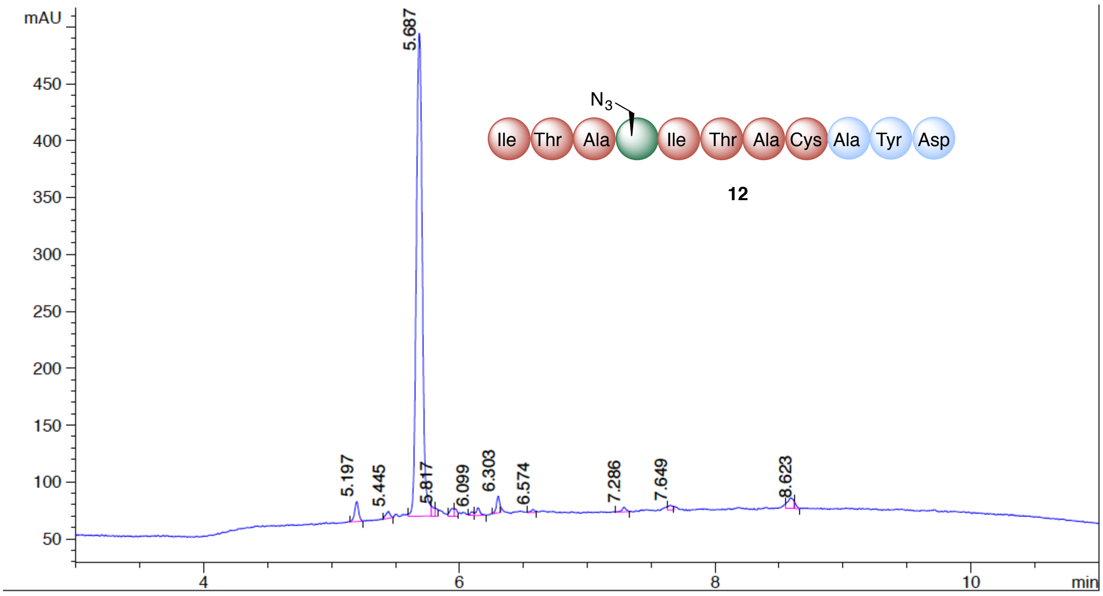

Figure S6: HPLC trace of peptide **12** carried out using a 130Å 4.6 x 50mm column, 10%-90% MeCN gradient (0.1% TFA; 1.5 mL/min), in 8 minutes at 60 °C.

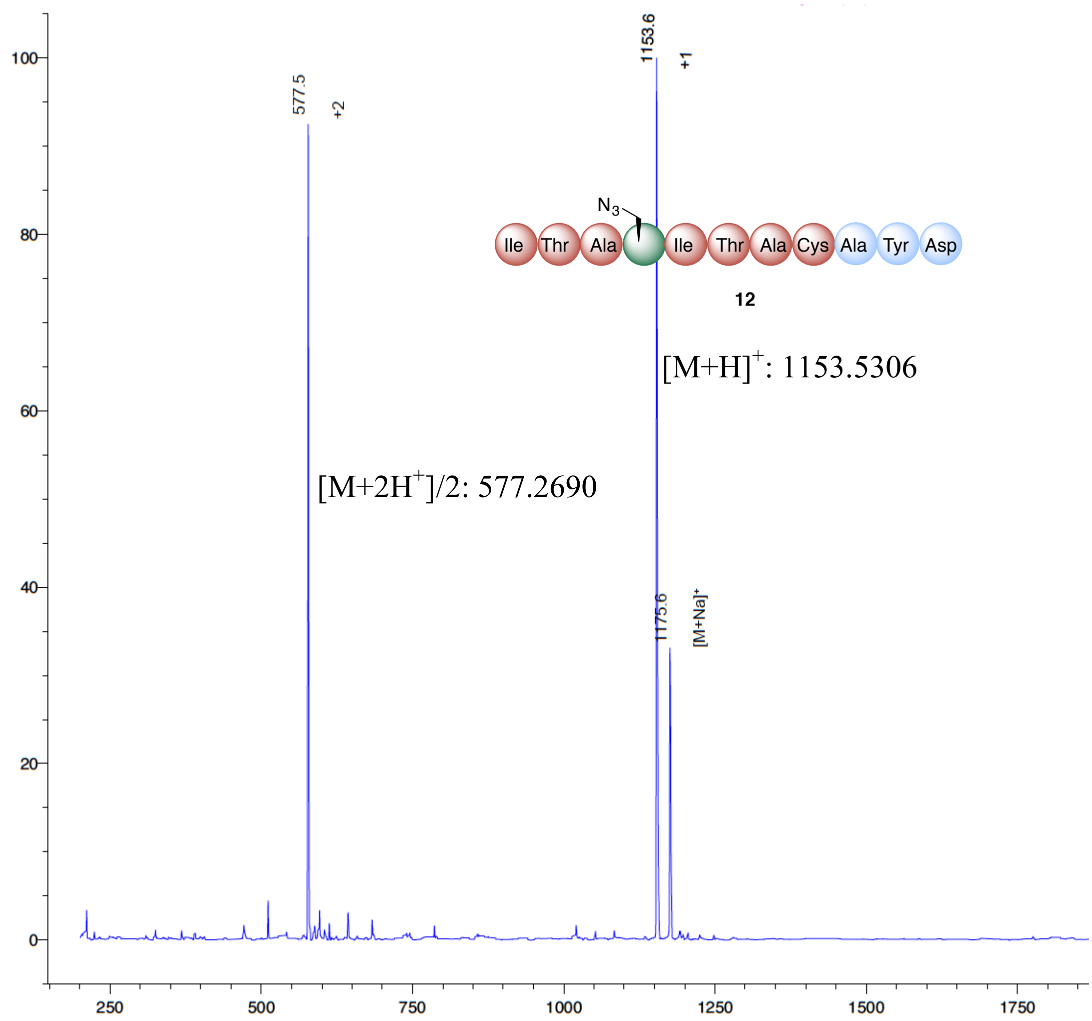

Figure S7: API-ESI$^+$ spectrum of peptide **12**.

III. Synthetic procedures and analysis data of cyclic peptides 2, 4, and 14

Cyclo(-ITAA(N_3)ITAP-) **2** was synthesized following procedure A using starting peptide **1**. HPLC purification (system P1) afforded the TFA salt of the desired compound as white solid (4.7 mg, 45%).

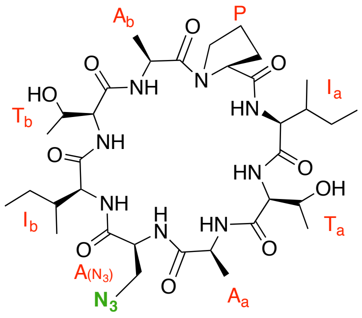

Cyclo(-$I_aT_aA_aA(N_3)I_bT_bA_bP$-)

^{1}H NMR: (500 MHz, DMSO); HSQC and HMBC analysis (500 MHz, DMSO)

Amino Acid*	Atome	^{1}H chemical shift	^{13}C chemical shift
Ile (a)	NH	8.14, d, $J = 7.1$	-
	α CH	4.22-4.19, m	57.3
	β CH	1.69, br, s	37.0
	γ CH_2	1.48-1.39, m; 1.02-0.98, m	24.5
	γ CH_3	0.88-0.79, m**	11.3
	δ CH_3	0.88-0.79, m**	11.3
	CO	-	170.9
Thr (a)	NH	8.00, d, $J = 4.2$	-
	α CH	3.96-3.90, m	59.5
	β CH	3.96-3.90, m	65.9
	γ CH_3	1.07, d, $J = 5.9$	20.0
	CO	-	ND
Ala (a)	NH	8.31, d, $J = 6.5$	-
	α CH	3.74-3.71, m	49.7
	β CH_3	1.37, d, $J = 7.1$	14.7
	CO	-	171.0
Ala(N_3)	NH	7.53, d, $J = 9.2$	-
	α CH	4.89, dd, $J = 7.2$, $J = 16.1$	50.5
	β CH_2	3.81-3.78, m; 3.54-3.44, m	52.7
	CO	-	170.1
Ile (b)	NH	8.65, d, $J = 6.4$	-
	α CH	4.07-4.05, m	59.0
	β CH	1.94-1.80, m	35.6
	γ CH_2	1.48-1.39, m; 1.27-1.28, m	24.8
	γ CH_3	0.88-0.79, m**	15.4
	δ CH_3	0.88-0.79, m**	15.4
	CO	-	169.8/171.6

Thr (b)	NH	7.45, d, *J* = 8.3	-
	α CH	4.07-4.05, m	59.0
	β CH	4.28-4.22, m	65.2
	γ CH_3	1.05, d, *J* = 6.4	20.7
	CO	-	169.8/171.6
Ala (b)	NH	7.12, d, *J* = 7.1	-
	α CH	4.58-4.52, m	46.3
	β CH_3	1.17, d, *J* = 6.6	17.4
	CO	-	172.1
Pro	α CH	4.47-4.45, m	60.6
	β CH_2	2.04-2.07, m; 1.94-1.80, m	26.7
	γ CH_2	1.94-1.80, m	24.5
	δ CH_2	3.51-3.55, m; 3.54-3.44, m	46.6
	CO	-	170.6

* CO corresponds the carbonyl group of the individual amino acid listed

** Unable to integrate and/or clearly assign the corresponding multiplet

IR (neat) cm^{-1}: 3283, 2922, 2114, 2077, 1647, 1522, 1136; Melting point: 200 °C *dec.*; MS (ESI+) m/z (%): 780.4 (100), 781.3 (40); MS 50% ^{15}N labelled (ESI+) m/z (%): 780.4 (100), 781.3 (40); HRMS (ESI+): Calc. for $C_{34}H_{57}N_{10}{}^{15}NO_{10}$ $[M+Na]^+$: 803.4152, found: 803.4166; Calc. for $C_{34}H_{57}N_{11}O_{10}$ $[M+Na]^+$: 802.4182, found: 802.4187; HPLC t_R = 25.86 (purity = 93%; System A1).

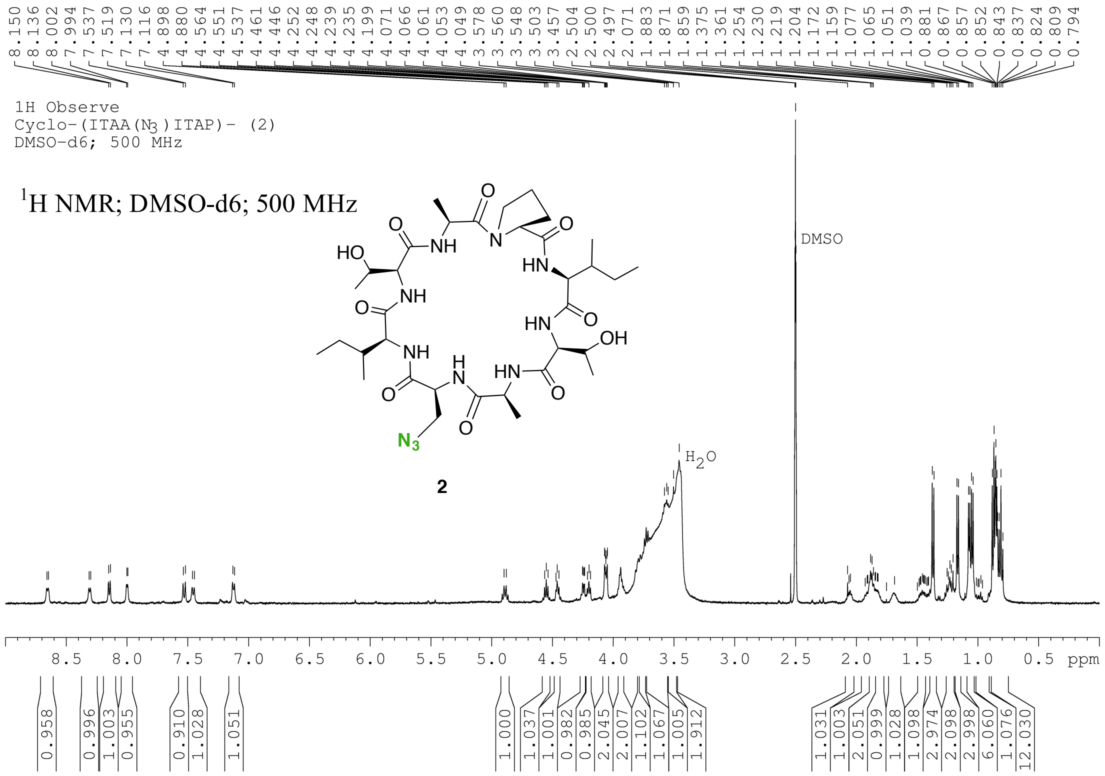

Figure S8: ^1H NMR spectrum of cyclic peptide **2**.

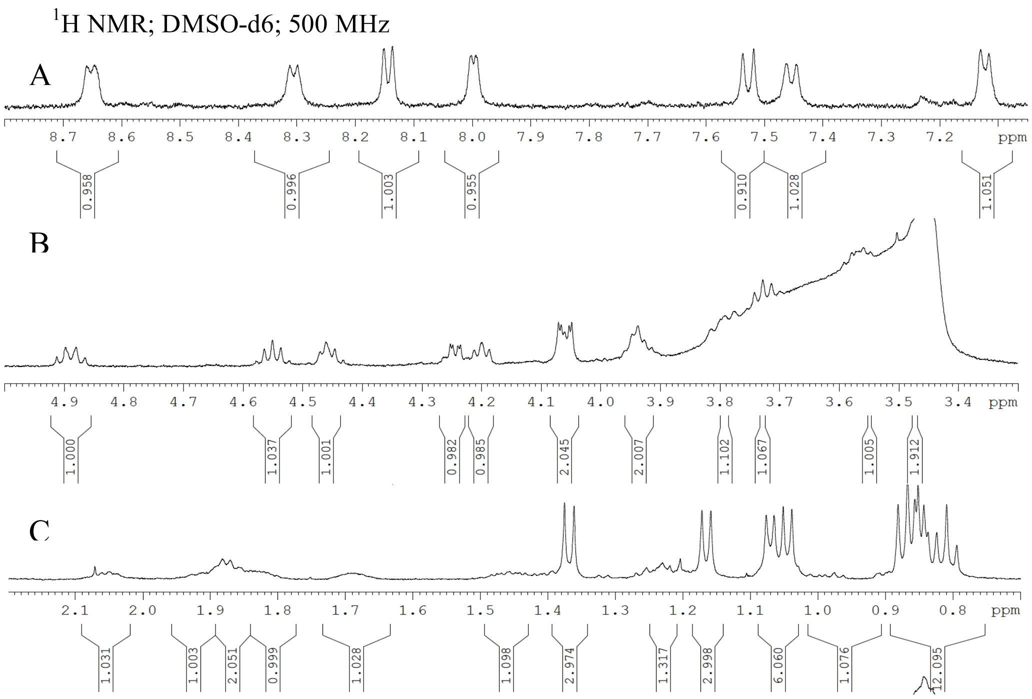

Figure S9: Detailed ^{1}H NMR spectrum of cyclic peptide **2**. A 8.8-7.1 ppm; B 5.0-3.3 ppm; C 2.2-0.7 ppm.

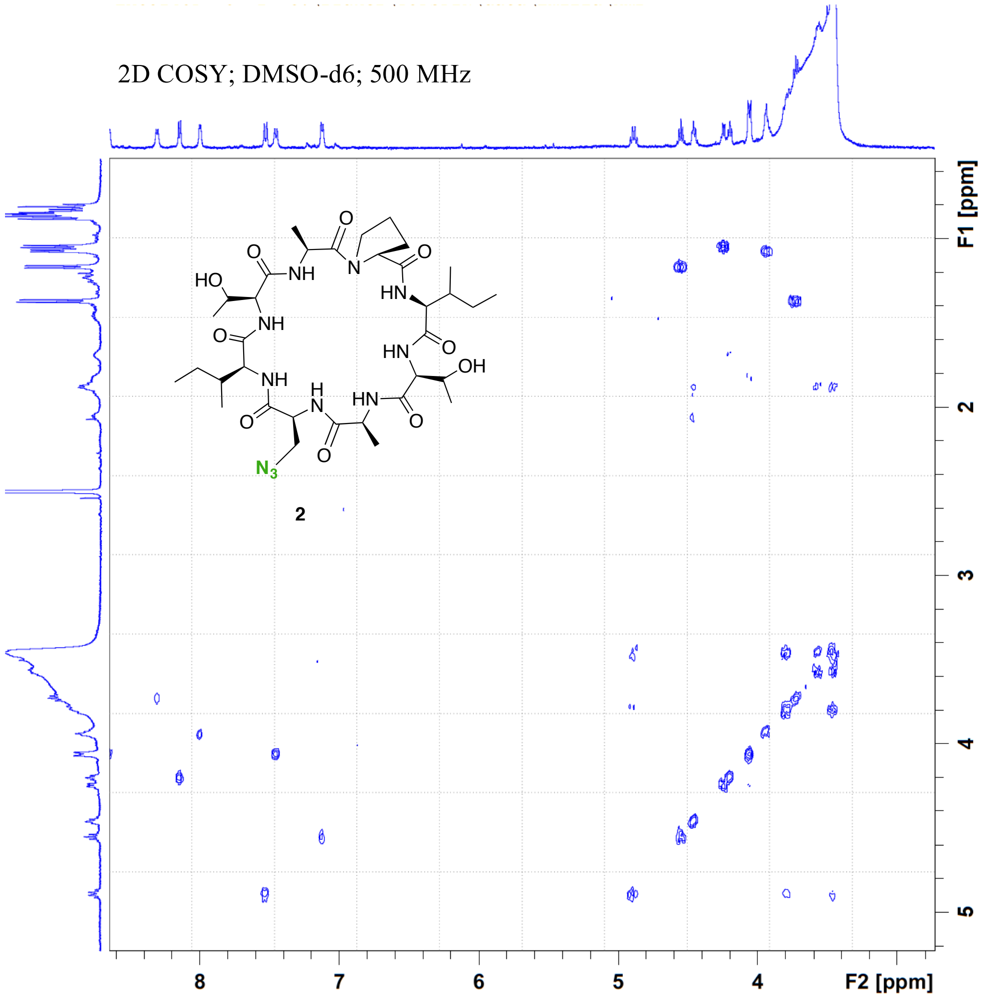

Figure S10: COSY spectrum of cyclic peptide **2**.

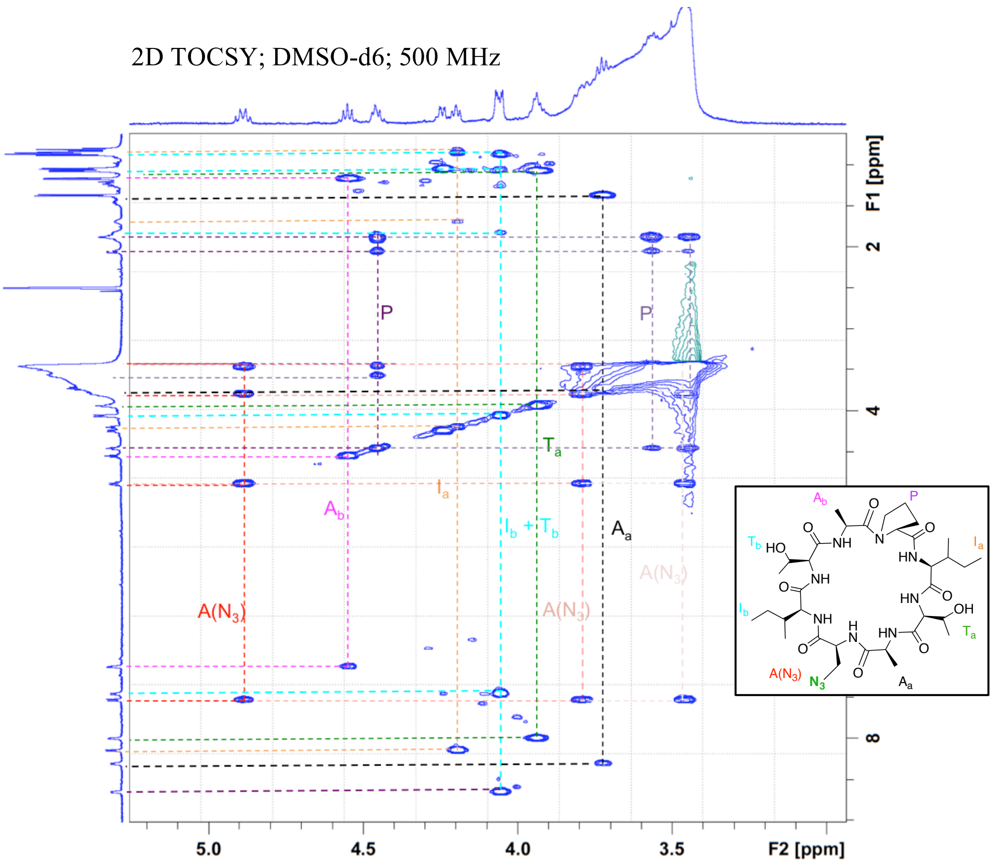

Figure S11: TOCSY spectrum of cyclic peptide **2**. Each individual amino acid has been color-coded and its corresponding TOCSY cross peaks linked to each other.

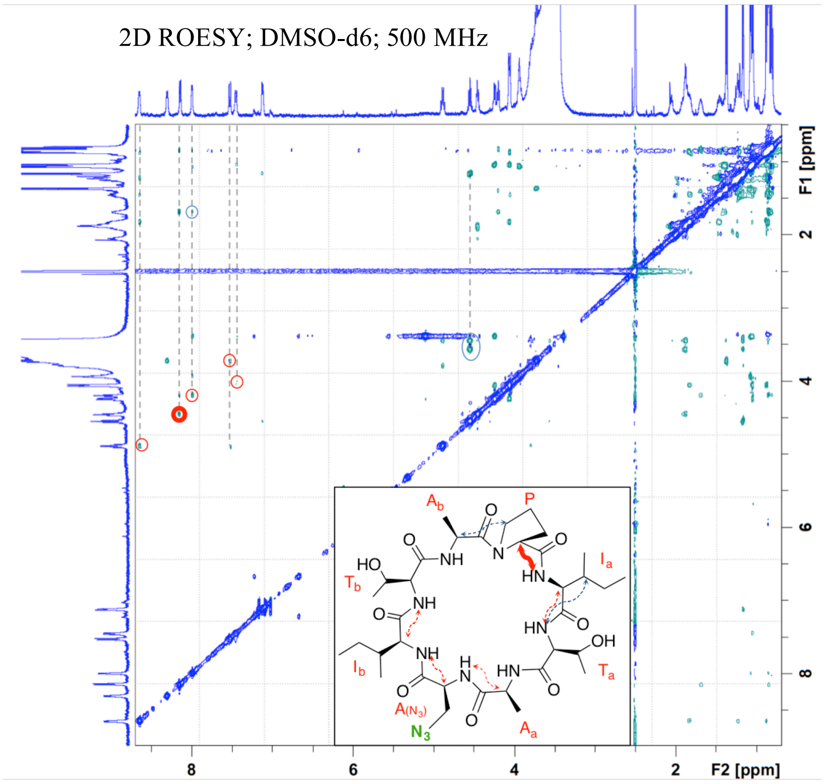

Figure S12: ROESY spectrum of cyclic peptide **2**. Arrow in bold proves the macrocycle formation

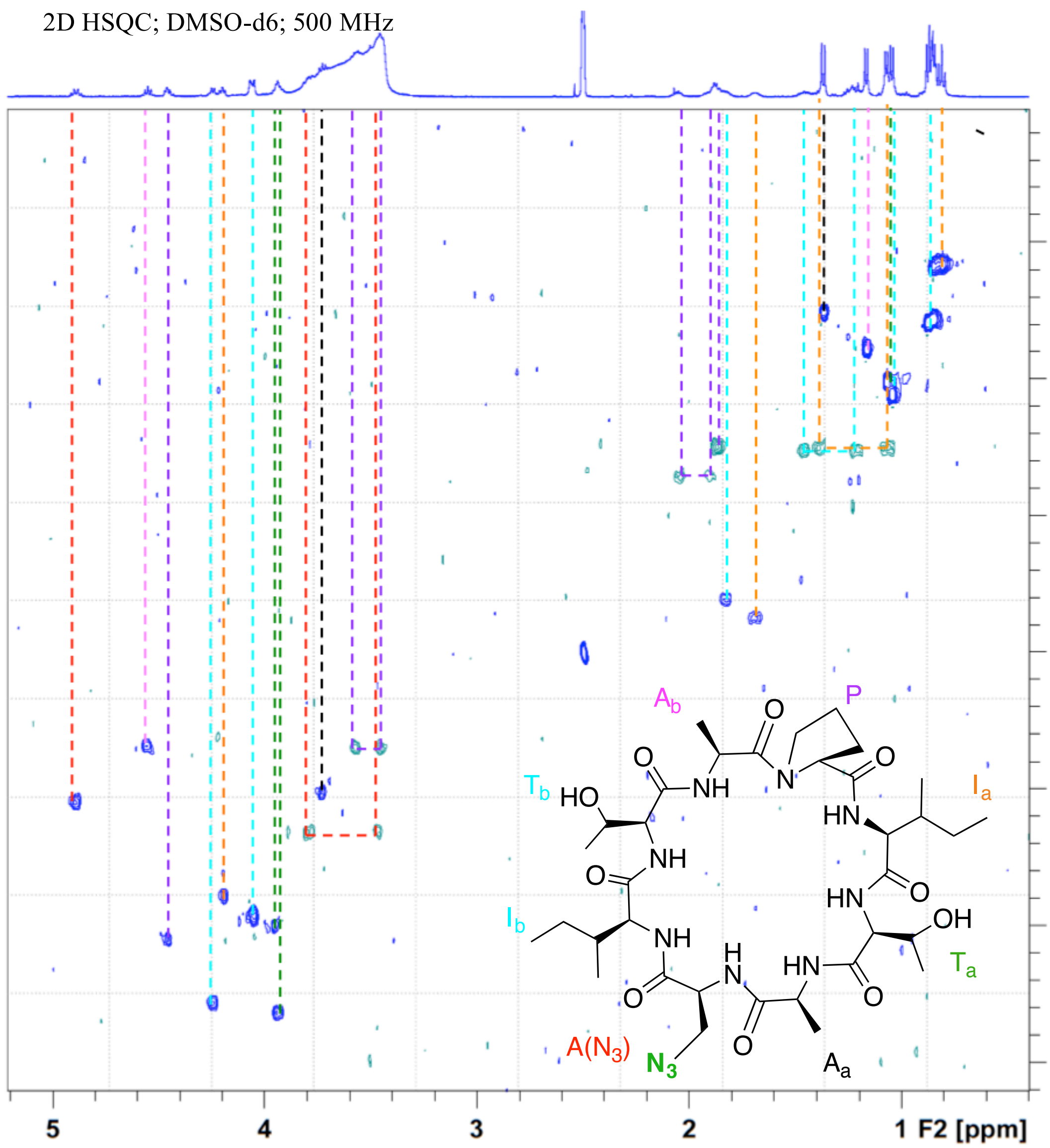

Figure S13: HSQC spectrum of cyclic peptide **2**. Each individual amino acid has been color-coded. CH and CH_3 carbons are in blue, CH_2 carbons are in green.

MSMS fragmentation data is essential to prove that the compound in hand is indeed a macrocycle. Contrary to linear peptides, the fragmentation of cyclic peptides can start at any point of the macrocycle before the chain continues to fragment. Hence, fragments containing both the N-terminal Ile and the C-terminal Pro can only exist in the fragmentation pattern of the cyclic peptide. Furthermore, CO losses are more common in cyclic peptides.

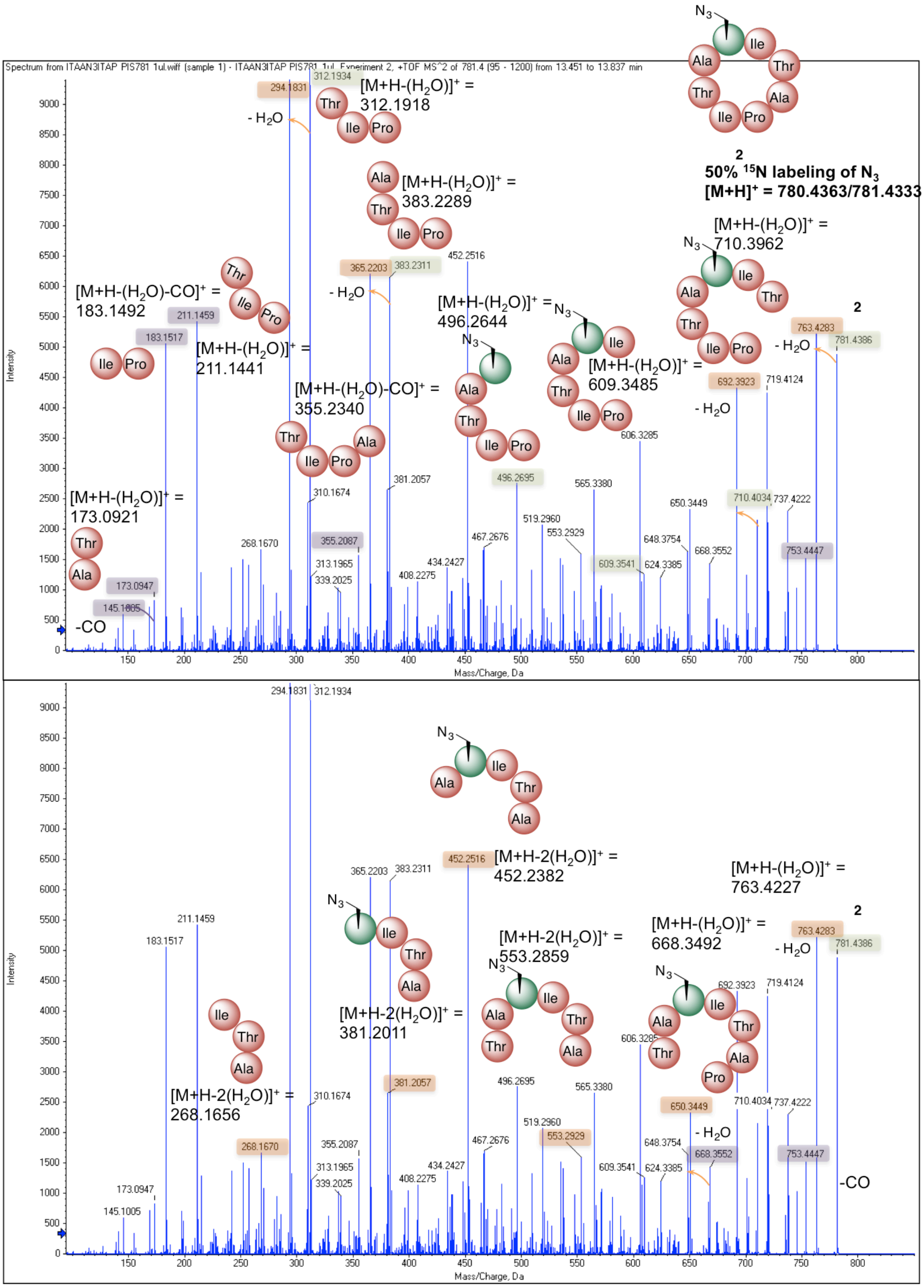

Figure S14. MSMS fragmentation data of cyclic peptide **2** on m/z = 781.4. The corresponding fragments and their theoretical mass are shown. The actual fragment masses are highlighted in green when the mass is derived from the loss of one or more amino acids; in orange when the mass is derived from the loss of an additional water (H_2O) molecule, and in purple when the mass is derived from the loss of an additional carbonyl (CO) group.

Cyclo(-VDhaAGIGFP-) **4** was synthesized following procedure A using peptide **3**. HPLC purification (system P2) afforded the ammonium salt of the desired compound as white solid (11.3 mg, 63%).

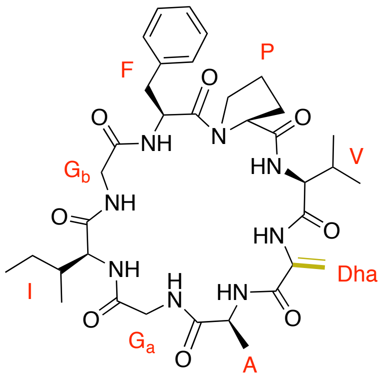

Cyclo(-VDhaAG$_a$IG$_b$FP-)

^{1}H NMR: (700 MHz, DMSO); HSQC and HMBC analysis (700 MHz, DMSO)

**Amino Acid***	**Atome**	**^{1}H chemical shift**	**^{13}C chemical shift**
Val	NH	8.60, d, J = 8.9	-
	α CH	4.06, dd, J = 6.4, J = 8.7	60.1
	β CH	2.29-2.24, m	28.4
	γ CH_3	0.82-0.79, m**	18.5/20.0
	CO	-	169.6
Dha	NH	8.50-8.49, m	-
	α C	-	133.4
	β CH_2	6.29, s; 5.74, s	102.5
	CO	-	170.6
Ala	NH	8.70, d, J = 6.9	-
	α CH	4.50-4.45, m	49.0
	β CH_3	1.38, d, J = 7.3	18.4
	CO	-	174.8
Gly (a)	NH	8.85, dd, J = 3.9, J = 5.9	-
	α CH_2	3.95, dd, J = 3.6, J = 14.7; 3.55, dd, J = 6.6, J = 14.4	43.8
	CO	-	172.7
Ile	NH	8.50-8.49, m	-
	α CH	4.00, t, J = 3.0	59.5
	β CH	1.87-1.82, m	35.7
	γ CH_2	1.40-1.26, m	24.6
	γ CH_3	0.92, d, J = 6.9	15.9
	δ CH_3	0.85, t, J = 7.40	11.9
	CO	-	170.6
Gly (b)	NH	7.95, d, J = 6.0	-
	α CH_2	3.86, dd, J = 7.5, J = 17.3; 3.17-3.14, m	41.9
	CO	4.28-4.22, m	170.0
Phe	NH	7.74, d, J = 1.0	-
	α CH	4.58-4.54, m	54.4
	β CH_2	3.26, dd, J = 4.2, J = 12.7;	37.7

		2.86, t, J = 12.2	
	Ar o-CH	7.24, t, J = 7.4	129.7
	Ar m-CH	7.36, t, J = 7.5	129.3
	Ar p-CH	7.30, t, J = 7.4	127.6
	Ar C	-	136.7
	CO	-	170.8
Pro	α CH	3.35-3.34, m	60.5
	β CH_2	1.87-1.82, m; 0.82-0.79, m	30.2
	γ CH_2	1.84-1.59, m; 1.40-1.26, m	21.8
	δ CH_2	3.35-3.34, m; 3.17-3.14, m	46.2
	CO	-	171.0

* CO corresponds the carbonyl group of the individual amino acid listed

** Unable to integrate and/or clearly assign the corresponding multiplet

IR (neat) cm^{-1}: 3281, 2967, 1638, 1541, 1369, 1201, 1132, 1047, 833; Melting point: 240 °C *dec.*; MS (ESI+) m/z (%): 711.3 (100), 712.3 (40); HRMS (ESI+): Calc. for $C_{35}H_{50}N_8O_8$ $[M+Na]^+$: 733.3644, found: 733.3628; HRMS (ESI-): Calc. for $C_{35}H_{50}N_8O_8$ $[M-H]^-$: 709.3679, found: 709.3680; HPLC t_R = 21.69 (purity > 95%; System A2).

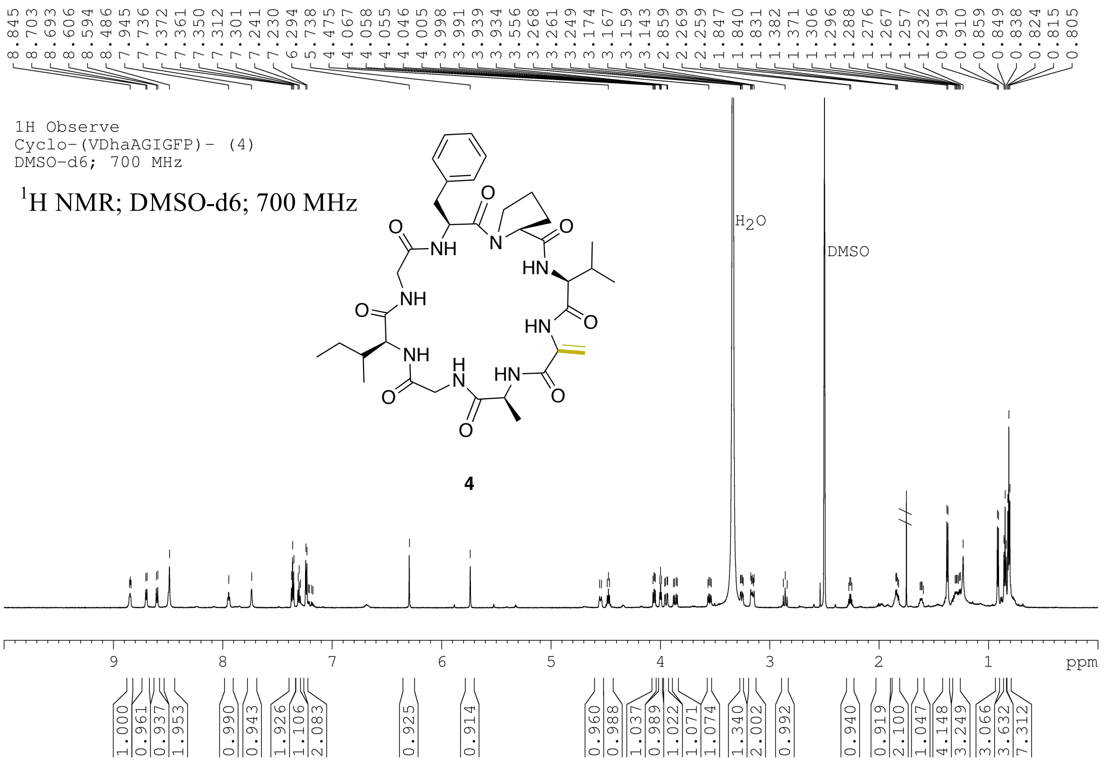

Figure S15: ^1H NMR spectrum of cyclic peptide **4**.

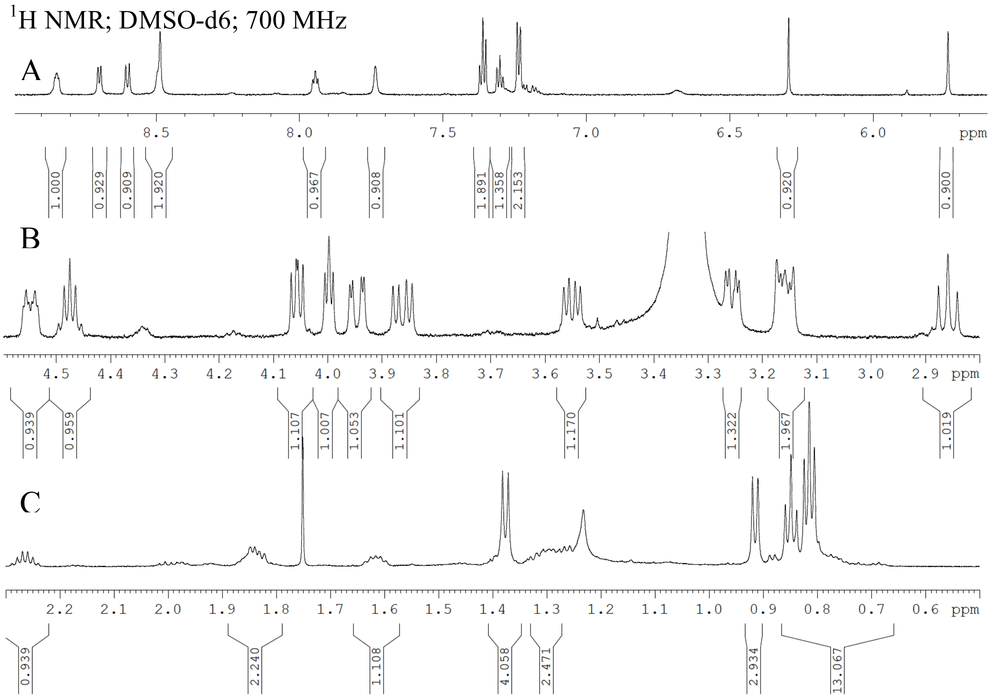

Figure S16: Detailed ^{1}H NMR spectrum of cyclic peptide **4**. A 8.9-5.6 ppm; B 5.0-2.8 ppm; C 2.3-0.5 ppm.

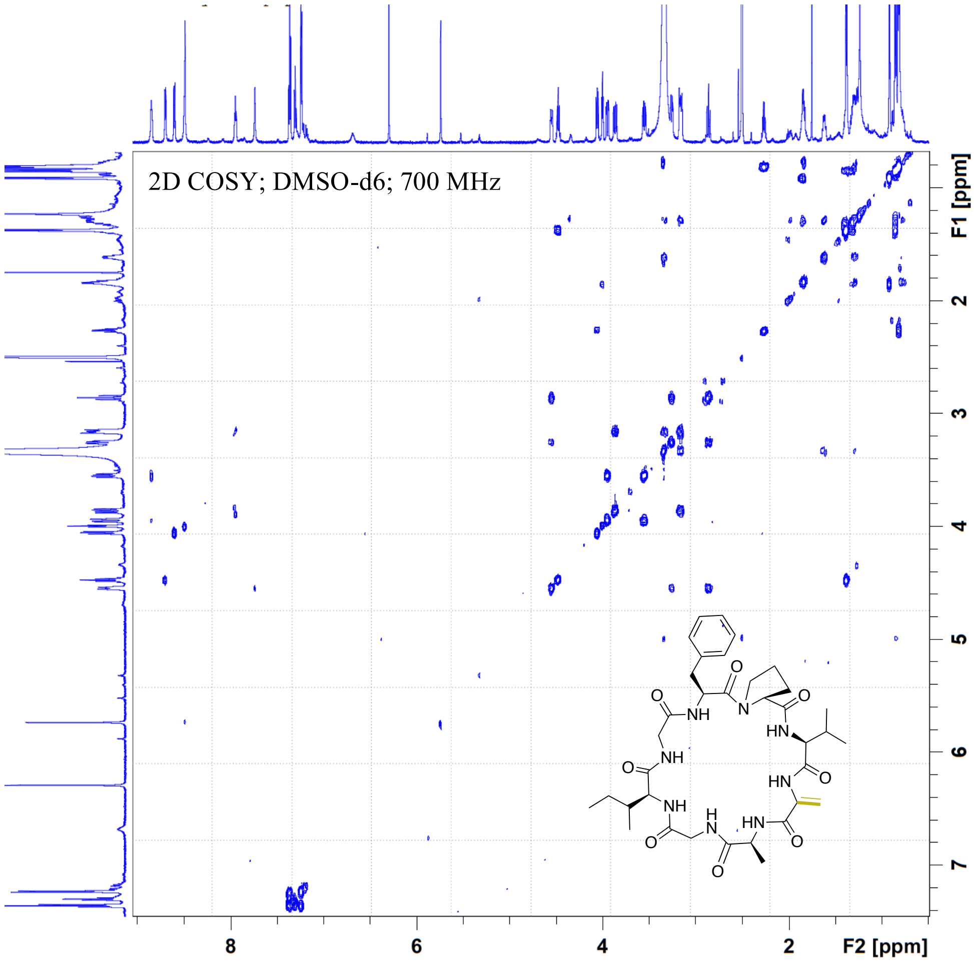

Figure S17: COSY spectrum of cyclic peptide **4**.

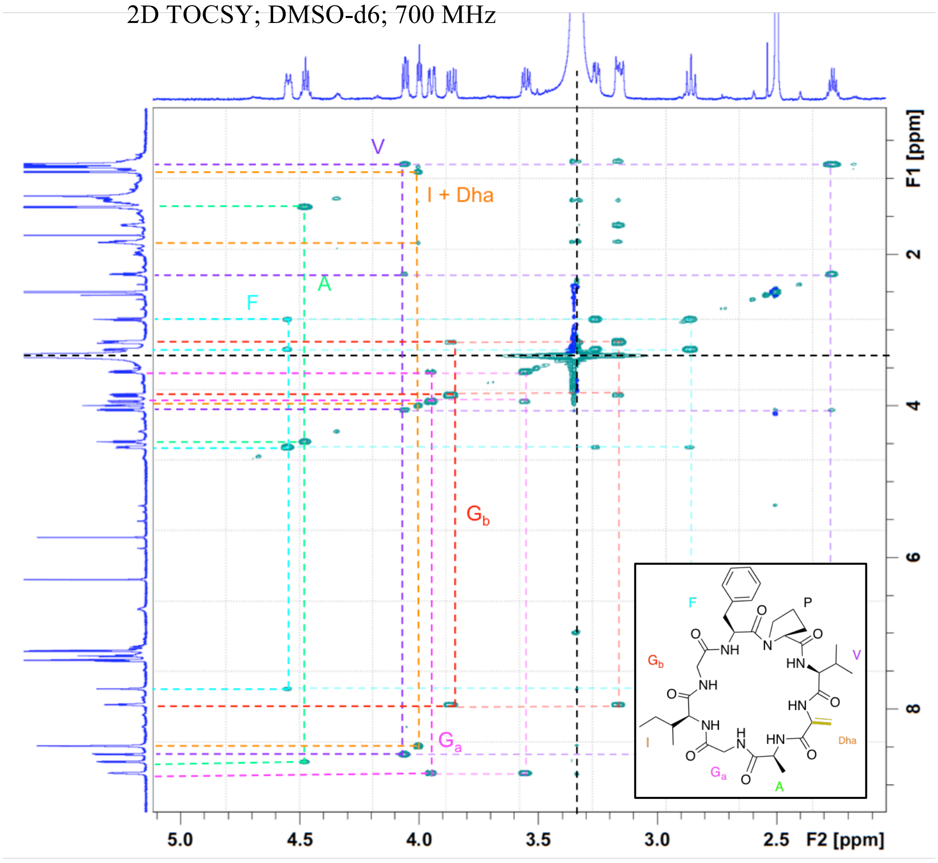

Figure S18: TOCSY spectrum of cyclic peptide **4**. Each individual amino acid has been color-coded and its corresponding TOCSY cross peaks linked to each other.

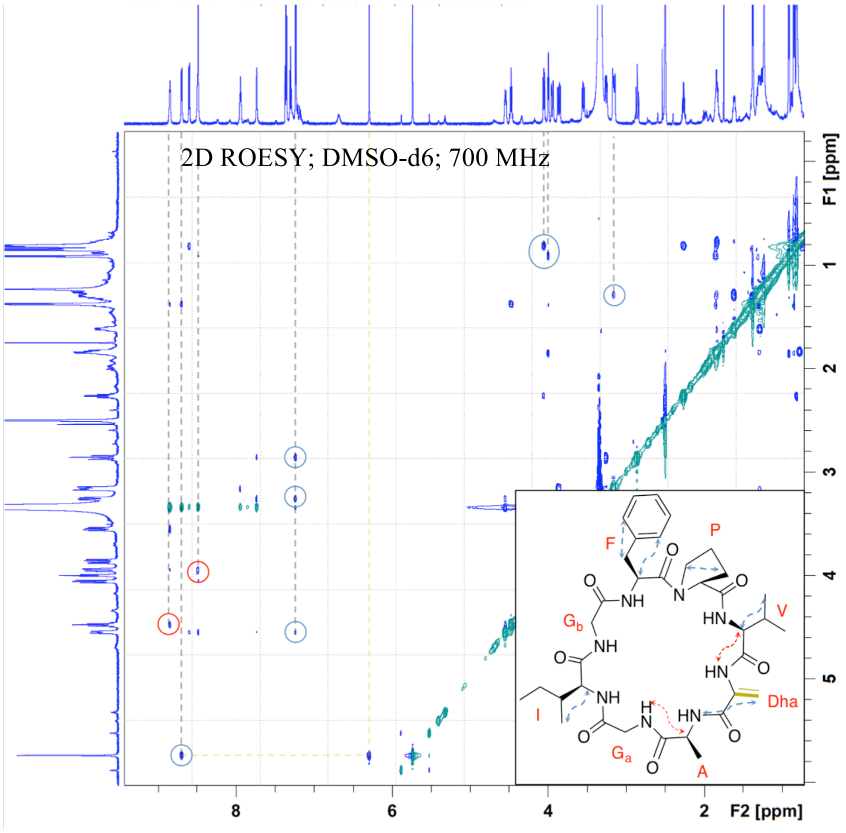

Figure S19: ROESY spectrum of cyclic peptide **4**.

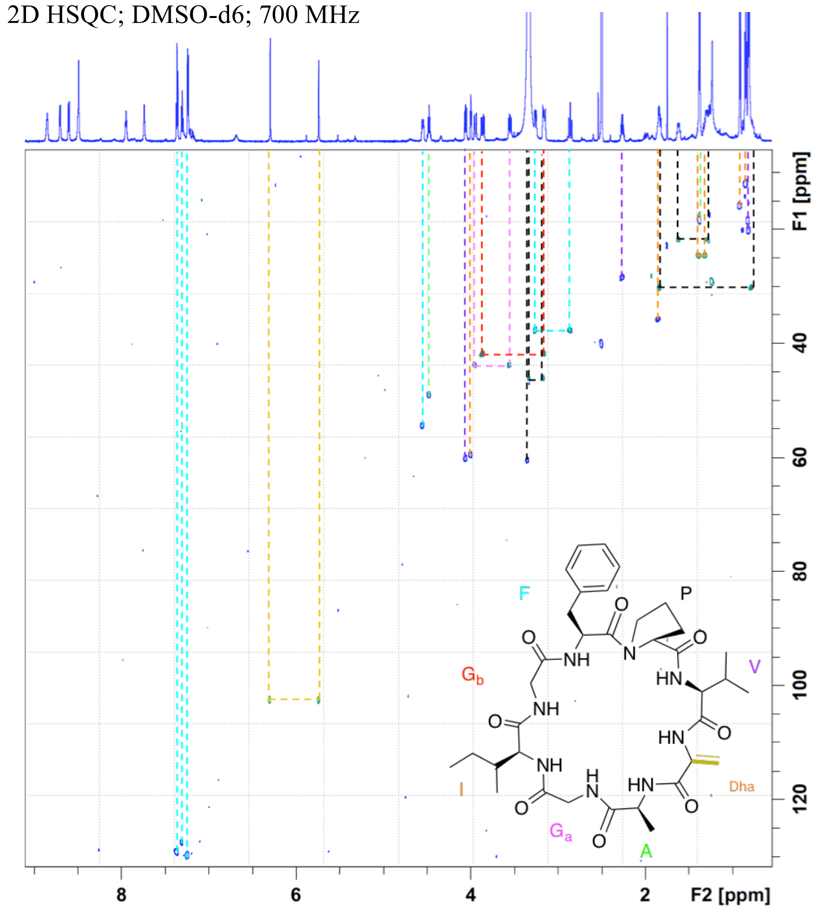

Figure S20: HSQC spectrum of cyclic peptide **4**. Each individual amino acid has been color-coded. CH and CH_3 carbons are in blue, CH_2 carbons are in green.

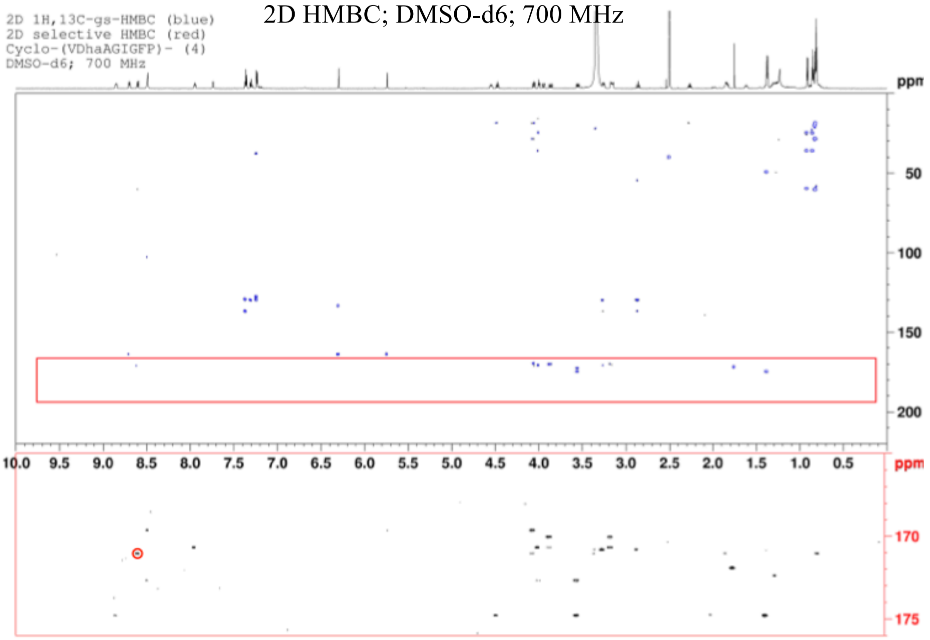

Figure S21: HMBC spectra of cyclic peptide **4**. Highlighted in red is the selective HMBC for the carbonyl region. The red spot is the cross peak between the NH of V and CO of P that proves the macrocycle formation

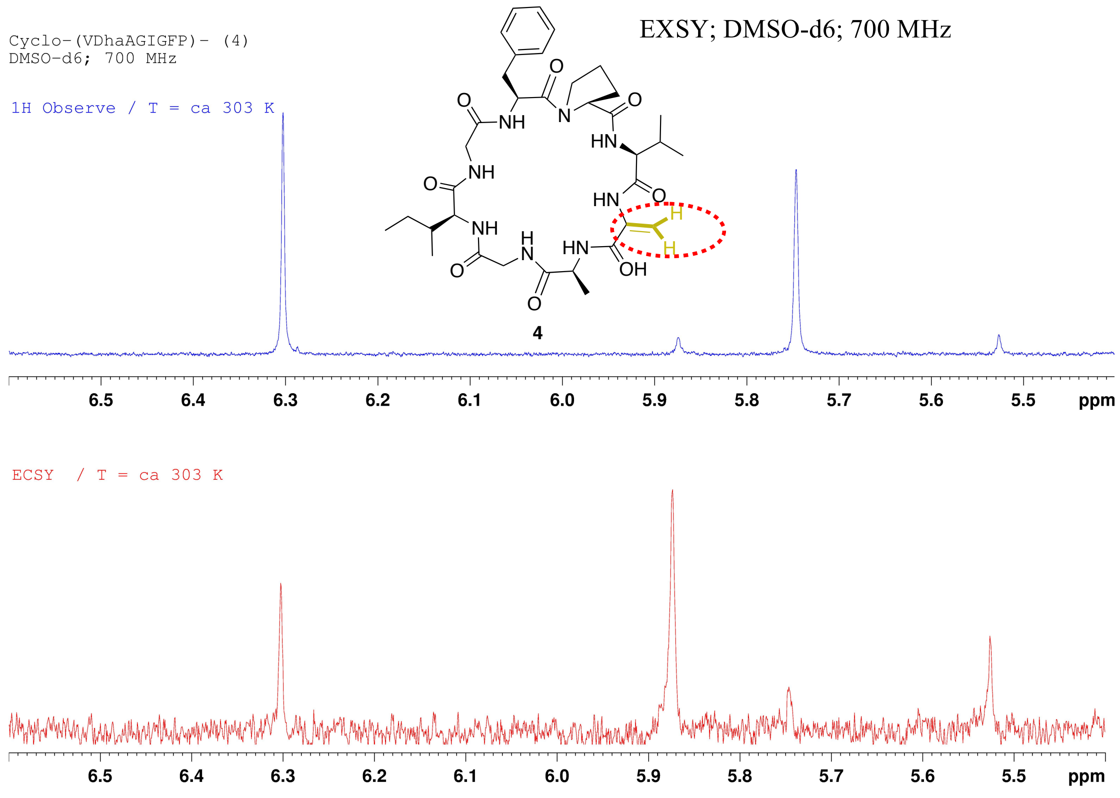

Figure S22: Comparison of normal 1H NMR spectrum (top) with EXSY spectrum (bottom) of cyclic peptide **4**. The major proton peaks of the CH_2 of Dha (blue) decrease in the EXSY experiment and the peaks of the other conformer (major red peaks) increase.

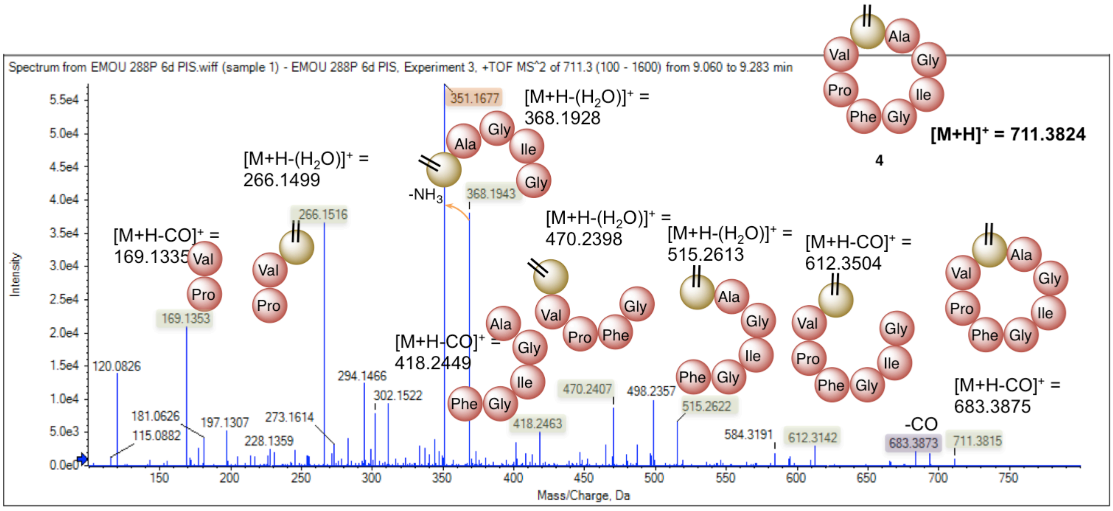

Figure S23. MSMS fragmentation data of cyclic peptide **4** on m/z = 711.4. The corresponding fragments and their theoretical mass are shown. The actual fragment masses are highlighted in green when the mass is derived from the loss of one or more amino acids; in orange when the mass is derived from the loss of an additional water (H_2O) molecule, and in purple when the mass is derived from the loss of an additional carbonyl (CO) group.

Cyclic peptide **14** was synthesized following procedure E. HPLC purification (system P2) afforded the desired compound as white solid (1.5 mg, 58%).

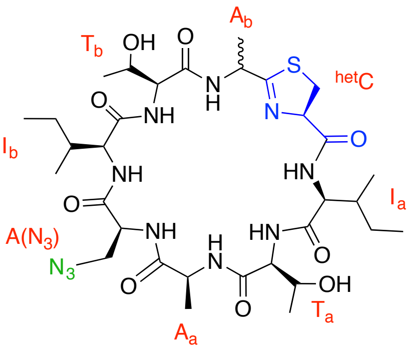

Cyclo(-$I_aT_aA_aA(N_3)I_bT_bA_b{}^{het}C$-)

^{1}H NMR: (500 MHz, DMSO); HSQC and HMBC analysis (700 MHz, DMSO)

Amino Acid*	Atome	^{1}H chemical shift	^{13}C chemical shift
Ile (a)	NH	7.32, br	-
	α CH	4.51-4.57, m	60.0
	β CH	1.87, br, s	36.2
	γ CH_2	1.35-1.30, m**	25.0
	γ CH_3	0.85-0.80 m**	16.1
	δ CH_3	0.92, d, $J = 6.7$	12.2
	CO	-	ND
Thr (a)	NH	8.45, br	-
	α CH	3.76, br	ND
	β CH	3.81-3.86, m	66.4
	γ CH_3	1.09, d, $J = 6.2$	20.9
	CO	-	ND
Ala (a)	NH	8.22, br	-
	α CH	3.90-3.93, m	50.0
	β CH_3	1.28, d, $J = 7.1$	17.1
	CO	-	172.1
Ala(N_3)	NH	7.77, br	-
	α CH	5.10, dd, $J = 4.7$, $J = 9.5$	78.0
	β CH_2	3.84-3.57, m	37.0
	CO	-	ND
Ile (b)	NH	8.49, br	-
	α CH	4.08-4.11, m	ND
	β CH	1.87, br, s	38.1
	γ CH_2	1.26-1.20, m; 1.43-1.37, m	24.5
	CH_3	0.76, t, $J = 7.3$	16.2
	CH_3	0.85-0.80, m**	11.4
	CO	-	ND
Thr (b)	NH	7.52, br	-
	α CH	4.02-4.08, m	59.2
	β CH	4.02-4.08, m	65.8
	γ CH_3	1.06, d, $J = 5.9$	21.4
	CO	-	ND

Ala (b)	NH	8.13, br	-
	α CH	4.67-4.73, m	48.2
	β CH_3	1.31, d, *J* = 7.0	20.2
	CN	-	176.3
^{het}C	α	4.51-4.57, m	ND
	β	3.57-3.48, m	51.1
	CO	-	ND

* CO corresponds the carbonyl group of the individual amino acid listed

** Unable to integrate and/or clearly assign the corresponding multiplet

IR (neat) cm^{-1}: 3306, 2918, 2851, 2110, 1645, 1520, 1132; Melting point: 220 °C *dec.*; MS (ESI+) m/z (%): 768.3 (100), 769.3 (40); HRMS (ESI+): Calc. for $C_{32}H_{53}N_{11}O_9S$ $[M+H]^+$: 768.3821, found: 768.3812; Calc. for $C_{32}H_{53}N_{11}O_9S$ $[M+Na]^+$: 790.3641, found: 790.3630; Calc. for $C_{32}H_{53}N_{11}O_9S$ $[M+H]^+$: 766.3680, found: 766.3676; HPLC t_R = 25.03 (purity = 98%; system A2).

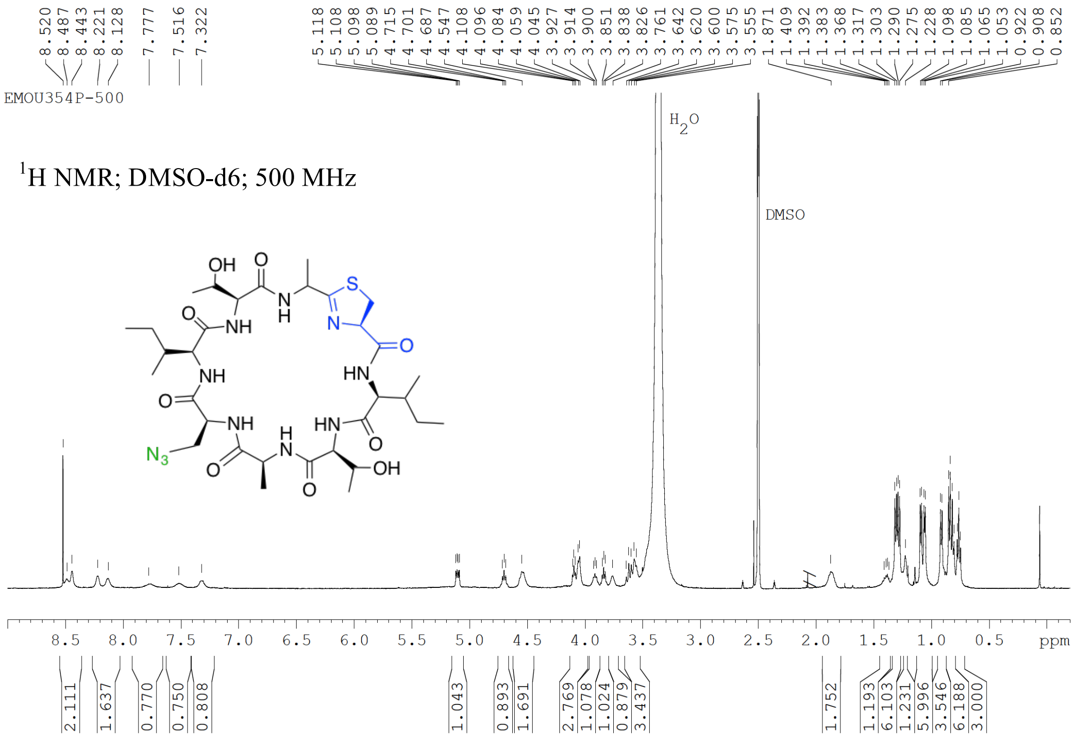

Figure S28: ^{1}H NMR spectrum of cyclic peptide **14**.

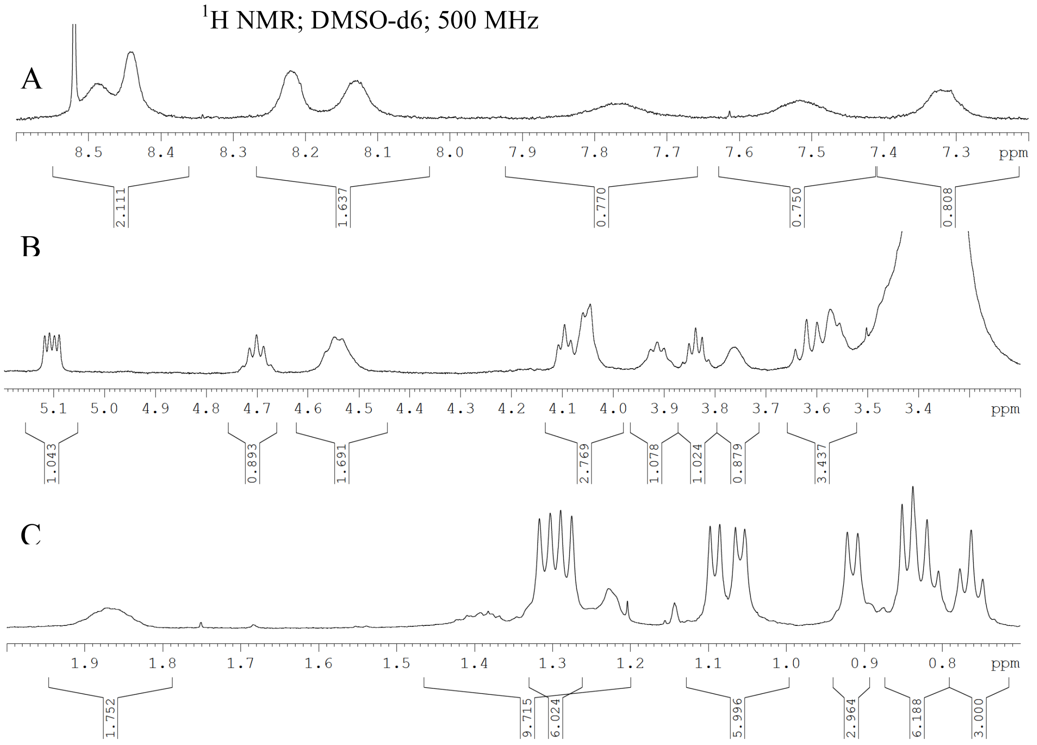

Figure S25: Detailed ^{1}H NMR spectrum of cyclic peptide **14**. A 8.6-7.2 ppm; B 5.2-3.2 ppm; C 2.0-0.7 ppm.

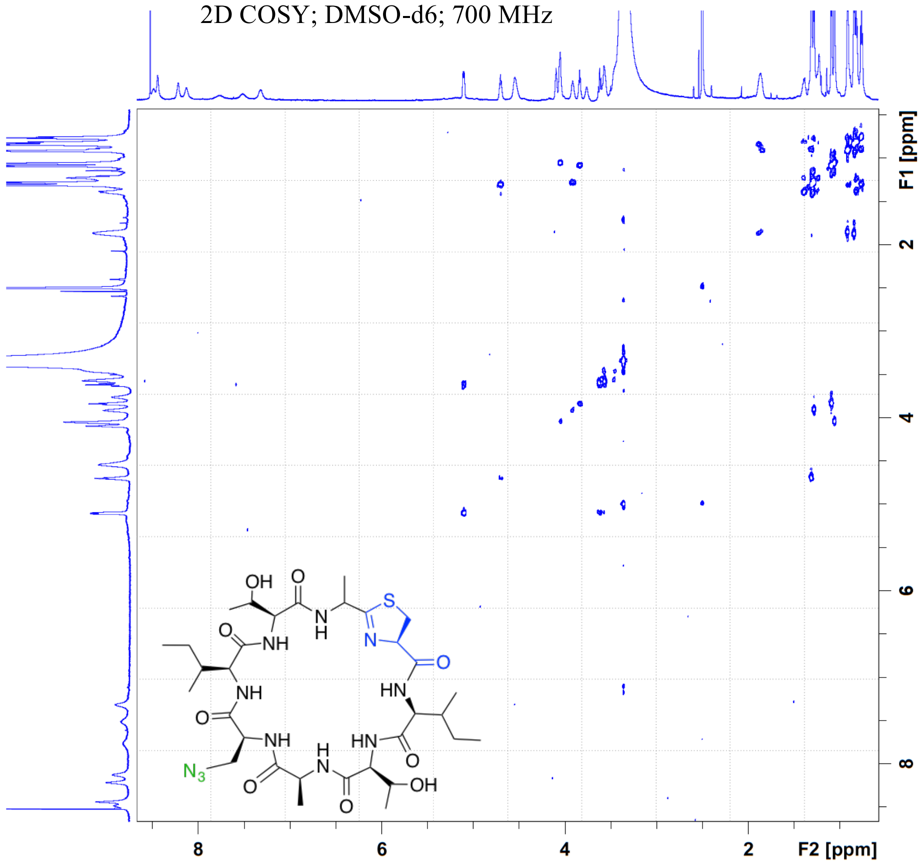

Figure S26: COSY spectrum of cyclic peptide **14**.

2D TOCSY; DMSO-d6; 700 MHz

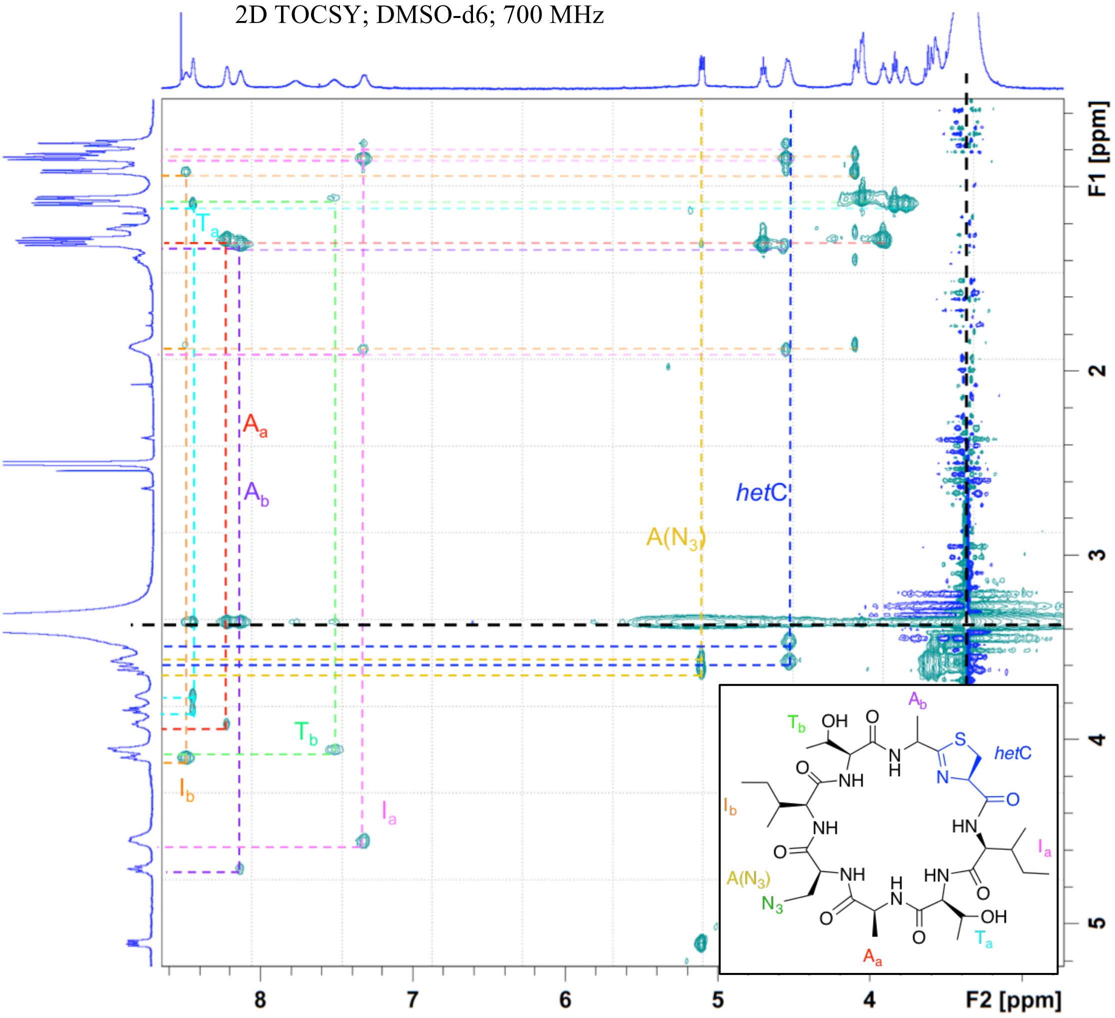

Figure S27: TOCSY spectrum of cyclic peptide **14**. Each individual amino acid has been color-coded and its corresponding TOCSY cross peaks linked to each other.

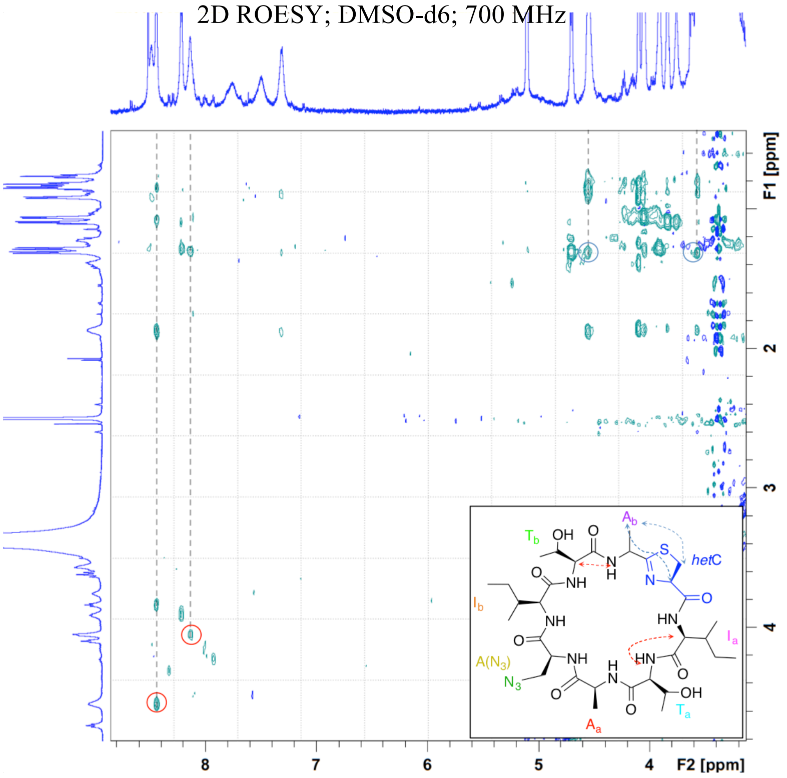

Figure S28: ROESY spectrum of cyclic peptide **14**.

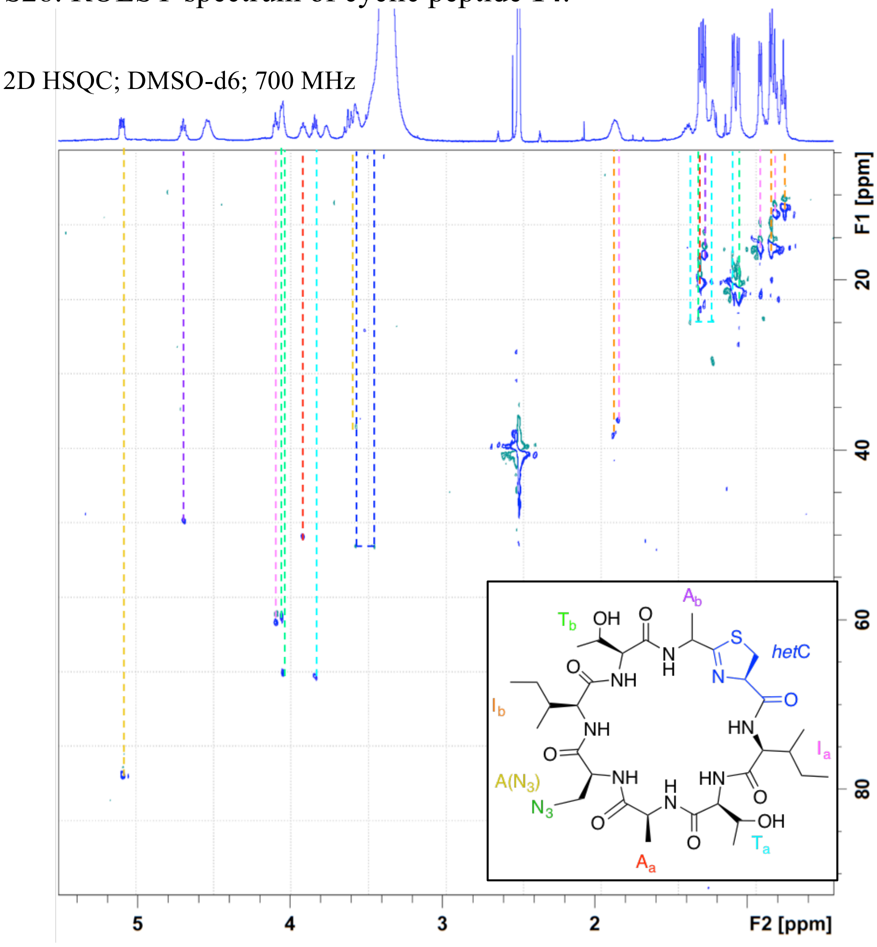

Figure S29: HSQC spectrum of cyclic peptide **14**. Each individual amino acid has been color-coded. CH and CH_3 carbons are in blue, CH_2 carbons are in green.

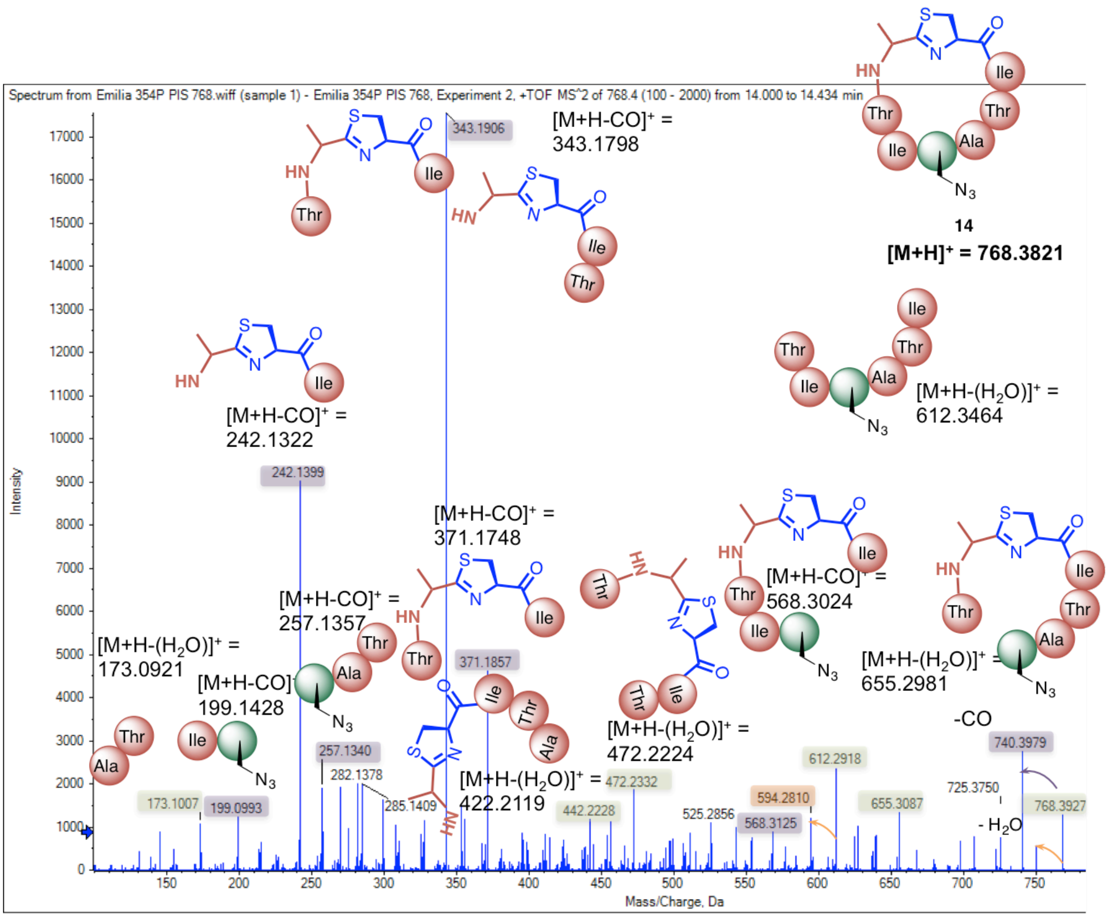

Figure S30. MSMS fragmentation data of cyclic peptide **14** on m/z = 768.4. The corresponding fragments and their theoretical mass are shown. The actual fragment masses are highlighted in green when the mass is derived from the loss of one or more amino acids; in orange when the mass is derived from the loss of an additional water (H_2O) molecule, and in purple when the mass is derived from the loss of an additional carbonyl (CO) group.

IV. Synthetic procedures of cyclic peptide derivatives 6, 8, 10, and 11

Triazole derivative **6** was synthesized following procedure B. HPLC purification (system P3, 200 nm) afforded the TFA salt of the desired compound as white solid (95%).

MS (ESI+) m/z (%): 930.5 (70), 931.5 (100), 932.5 (50); HRMS (ESI+): Calc. for $C_{44}H_{71}N_{11}O_{11}$ $[M+H]^+$: 930.5407, found: 930.5407; HRMS (ESI+): Calc. for $C_{44}H_{71}N_{10}{}^{15}NO_{11}$ $[M+H]^+$: 931.5378, found: 931.5392; HPLC t_R = 25.41 (purity > 92%; system A1).

Triazole derivative **8** was synthesized following procedure B. HPLC purification (system P3, 600 nm) afforded the TFA salt of the desired compound as blue solid (30%).
MS (ESI+) m/z (%): 895.0 (100), 895.4 (40); HRMS (ESI+): Calc. for $C_{86}H_{115}N_{15}O_{21}S_3$ $[M+H]^{2+}/2$: 894.8772, found: 894.8772; HRMS (ESI-): Calc. for $C_{86}H_{111}N_{15}O_{21}S_3$ $[M]^{2-}/2$: 892.8626, found: 892.8637; $HPLC_{600\,nm}$ t_R = 39.04 (purity > 95%; system A3).

Cyclic peptide **10** was synthesized following procedure C. HPLC purification (system P1) afforded the TFA salt of the desired compound as white solid (43%).
MS (ESI+) m/z (%): 1018.4 (100), 1019.3 (40); HRMS (ESI+): Calc. for $C_{45}H_{67}N_{11}O_{14}S$ $[M+H]^+$: 1018.4662, found: 1018.4660; Calc. for $C_{45}H_{67}N_{11}O_{14}S$ $[M+Na]^+$: 1040.4482, found: 1040.4479; HPLC t_R = 22.87 (purity = 93%; system A1).

Cyclic peptide **11** was synthesized following procedure D. HPLC purification (system P4) afforded two compounds a and b at different retention times bearing the same molecular weight (60%).
Compound a: MS (ESI+) m/z (%): 789.3 (100), 790.2 (40); HRMS (ESI+): Calc. for $C_{37}H_{56}N_8O_9S$ $[M+H]^+$: 789.3964, found: 789.3964; Calc. for $C_{37}H_{56}N_8O_9S$ $[M+Na]^+$: 811.3783, found: 811.3780; HRMS (ESI-): Calc. for $C_{37}H_{56}N_8O_9S$ $[M-H]^-$: 787.3818, found: 787.3823; HPLC t_R = 24.98 (purity = 90%; System A1).
Compound b: MS (ESI+) m/z (%): 789.3 (100), 790.2 (40); HRMS (ESI+): Calc. for $C_{37}H_{56}N_8O_9S$ $[M+H]^+$: 789.3964, found: 789.3959; Calc. for $C_{37}H_{56}N_8O_9S$ $[M+Na]^+$: 811.3783, found: 811.3773; HRMS (ESI-): Calc. for $C_{37}H_{56}N_8O_9S$ $[M-H]^-$: 787.3818, found: 787.3822; HPLC t_R = 26.18 (purity = 90%; System A1).

V. Additional figures

The macrocyclsation reaction was monitored at pH = 8.1 with the Dha peptide for a prolonged incubation period. Satisfyingly, we did not see any derivatization of the protein (Figure S31) via the Dha peptide. On the other hand, *tris*(2-carboxyethyl)phosphine (TCEP) which had been added to PatG_{mac} during

purification and storage, did react with Dha residue both in the starting material and cyclic peptides. The phosphine attacks the carbon double bond to generate the phosphonium moiety (Figure S32). Dialysis of the enzyme prior to the reaction to remove TCEP eliminated this side reaction.

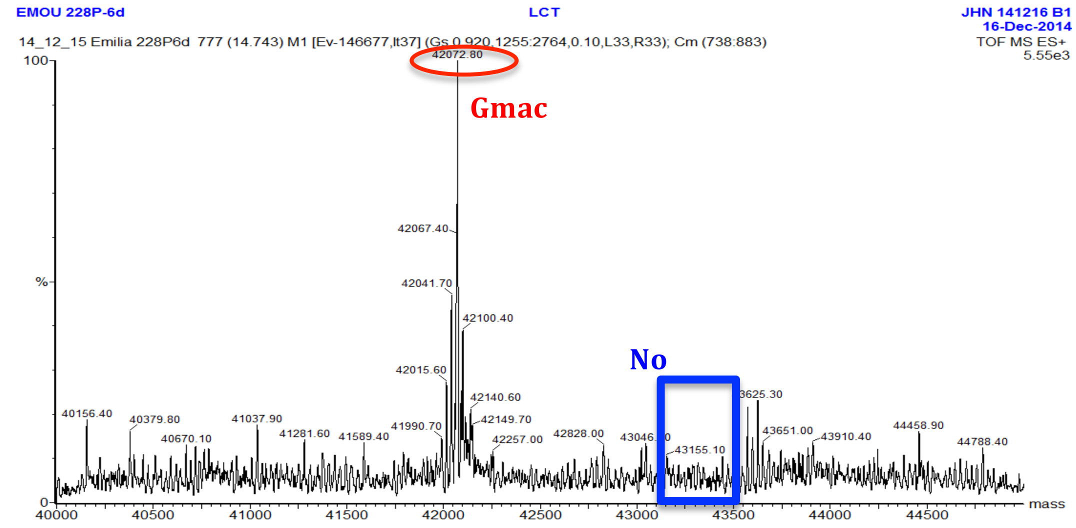

Figure S31. Maldi trace of the Gmac enzymatic reaction of **2** showing the Gmac deconvoluted peak whereas no peaks were detected for the Dha-enzyme adduct peak; expected mass: 43228.

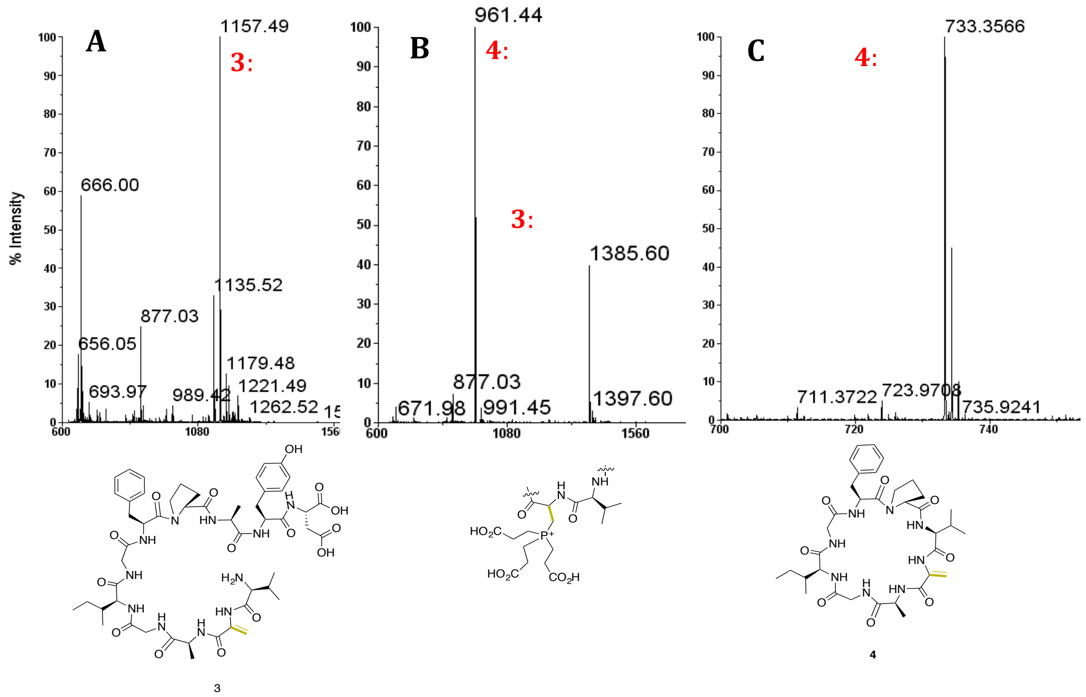

Figure S32. Maldi traces of: A- Linear peptide **3** before the addition of PatGmac; B- Incubation of the linear peptide **3** with PatGmac in the presence of TCEP; C- Incubation of the linear peptide **3** with PatGmac in the absence of TCEP

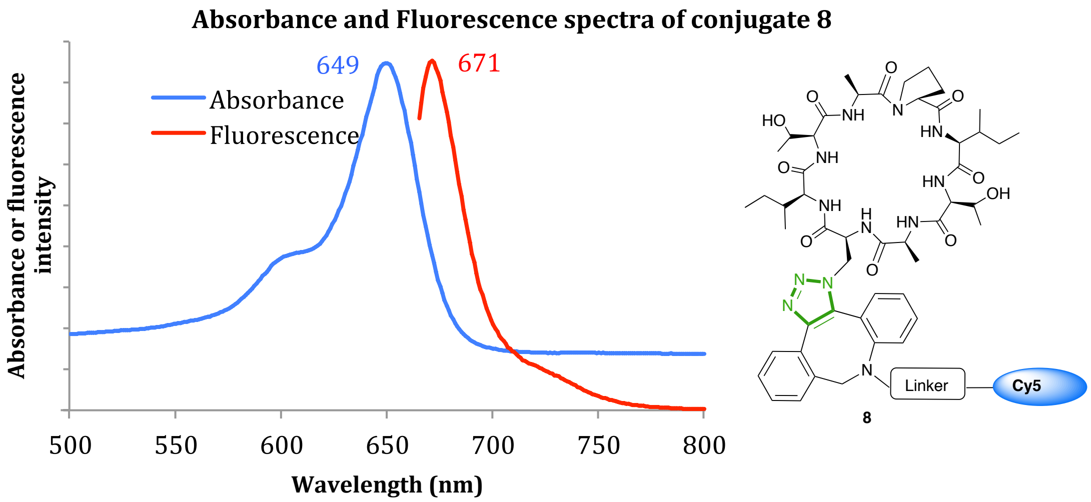

Figure S33. Normalised absorption (blue) and emission (red, λ_{ex} 649 nm, 665 nm cut off) spectra of conjugate **8**; (Abs/Em of DBCO-Cy5 **7** = 646/661 nm; Abs/Em of Cy5 = 646/670 nm).

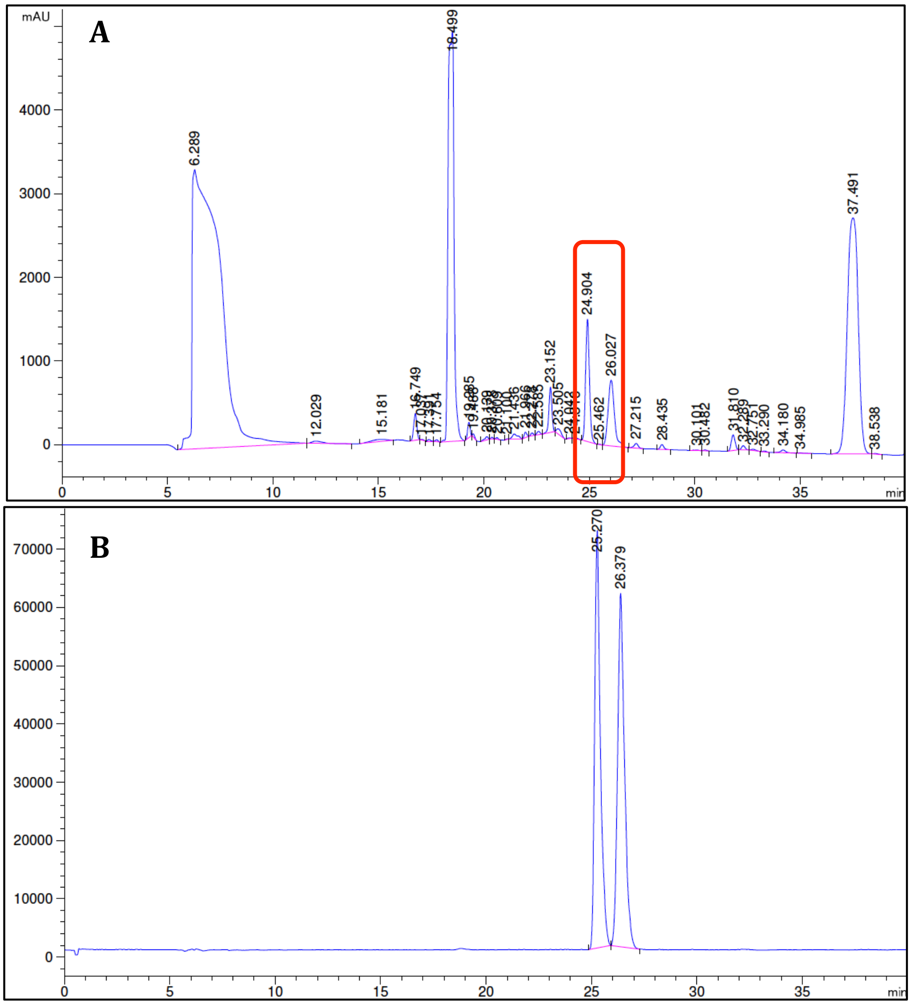

Figure S34: LCMS trace of the reaction mixture of cylic peptide **11**A- UV trace (220 nm); B- ESI$^+$-SIM mode trace at 789.4.

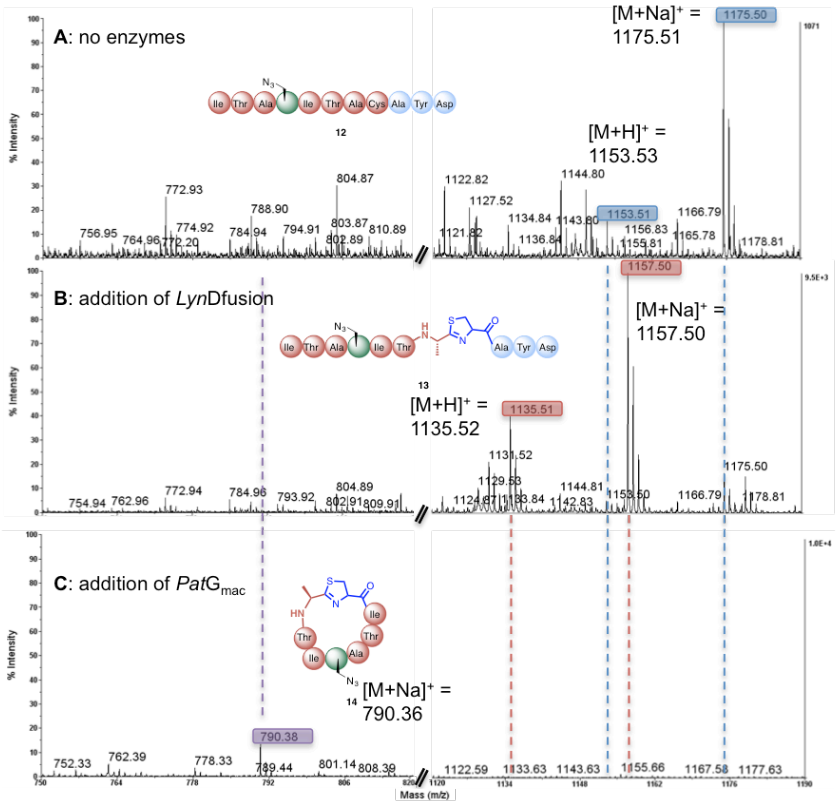

Figure S35. Maldi traces of the one-pot reaction: A- Starting peptide **12**; B- Overnight reaction with *Lyn*Dfusion; C- 4 days after the addition of *Pat*G_{mac}.

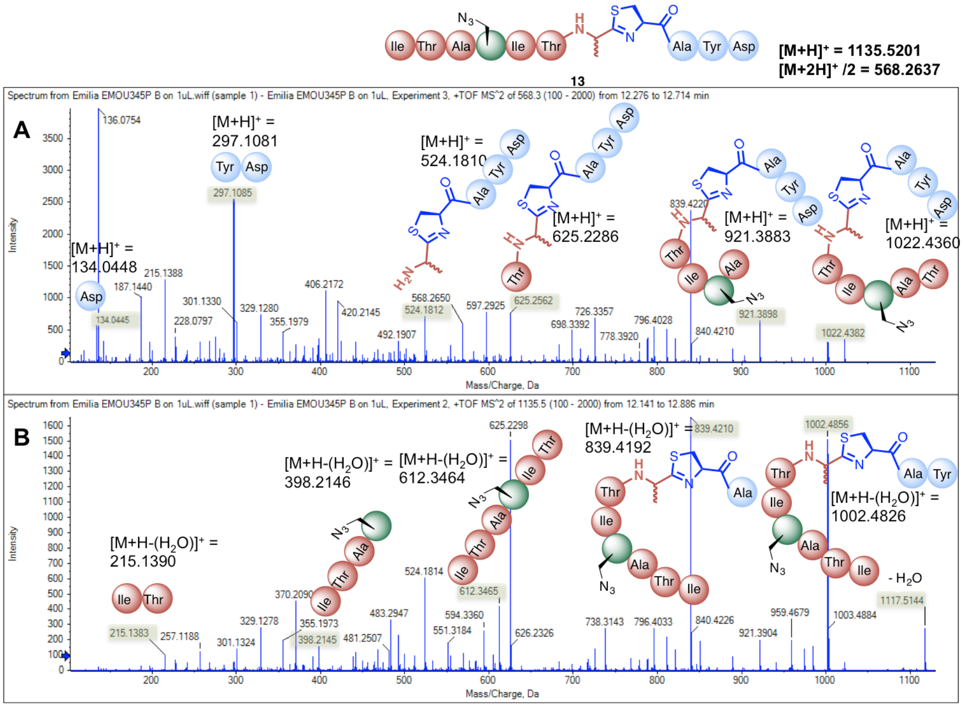

Figure S36. MSMS fragmentation data of intermediate peptide **13**; A- MSMS triggered on the doubly charged mass $[M+2H]^{+}/2 = 568.3$; B- MSMS triggered on the mono charged mass $[M+H]^{+} = 1135.5$. The corresponding fragments and their theoretical mass are shown. The actual fragment masses are highlighted.

VI. HPLC traces of compounds 2, 4, 6, 8, 10, 11, and 14

The HPLC methods used are described in paragraph I.

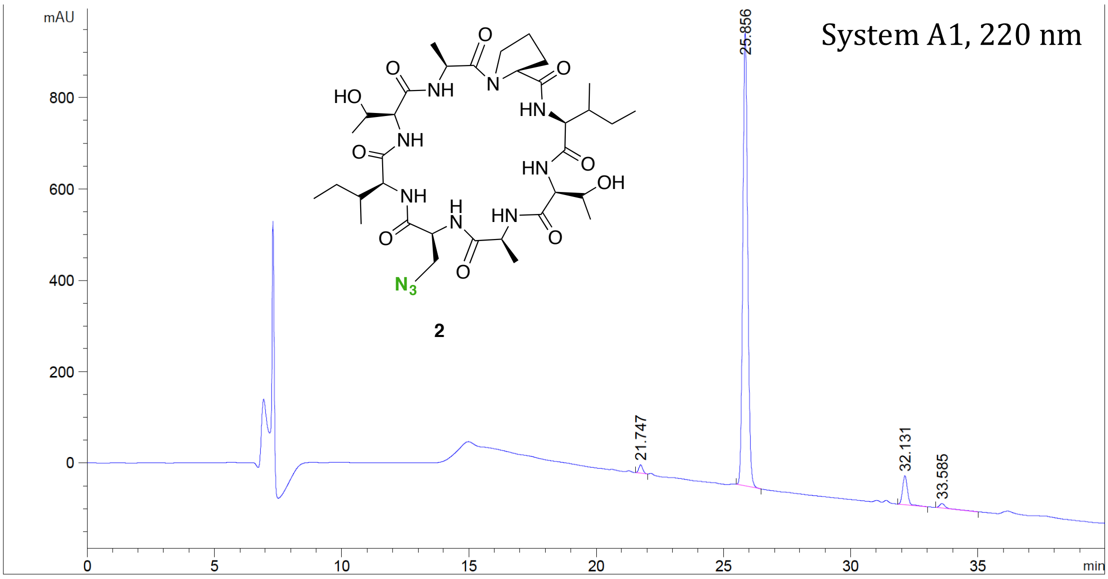

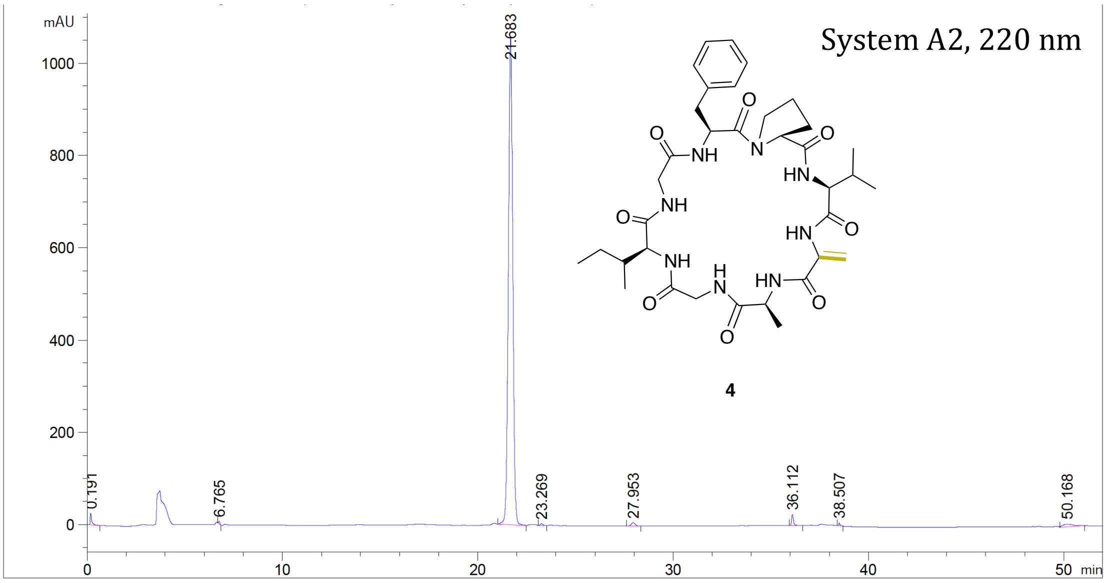

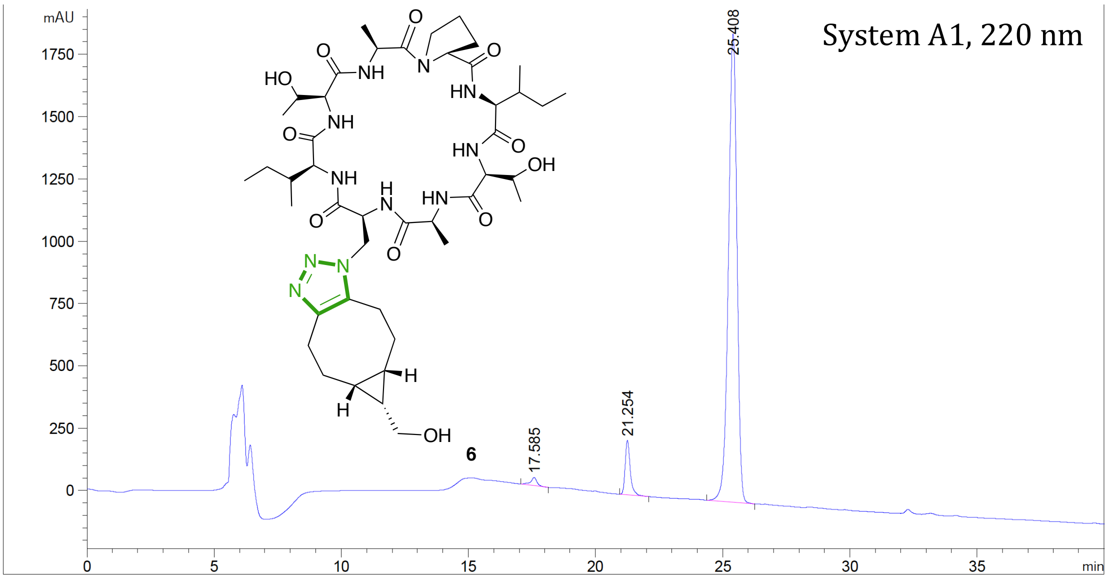
System A1, 220 nm
mAU
1750
1500
1250
1000
750
500
250
0
25.408
21.254
17.585
6
0
5
10
15
20
25
30
35
min

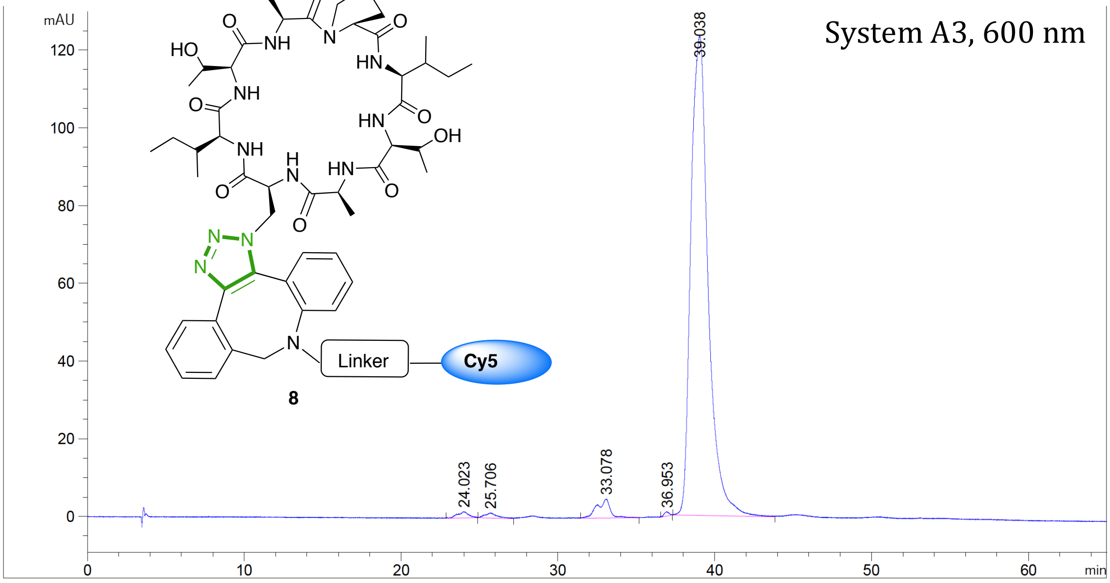
System A3, 600 nm
mAU
120
100
80
60
40
20
0
39.038
24.023
25.706
33.078
36.953
Linker
Cy5
8
0
10
20
30
40
50
60
min

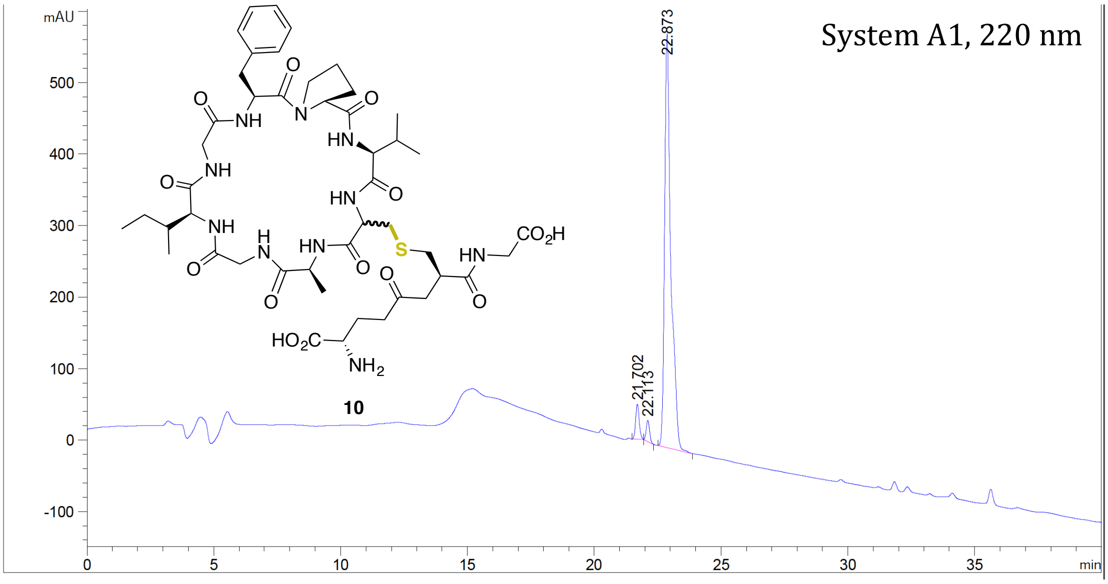
System A1, 220 nm
mAU
500
400
300
200
100
0
-100
22.873
21.702
22.113
10
0
5
10
15
20
25
30
35
min

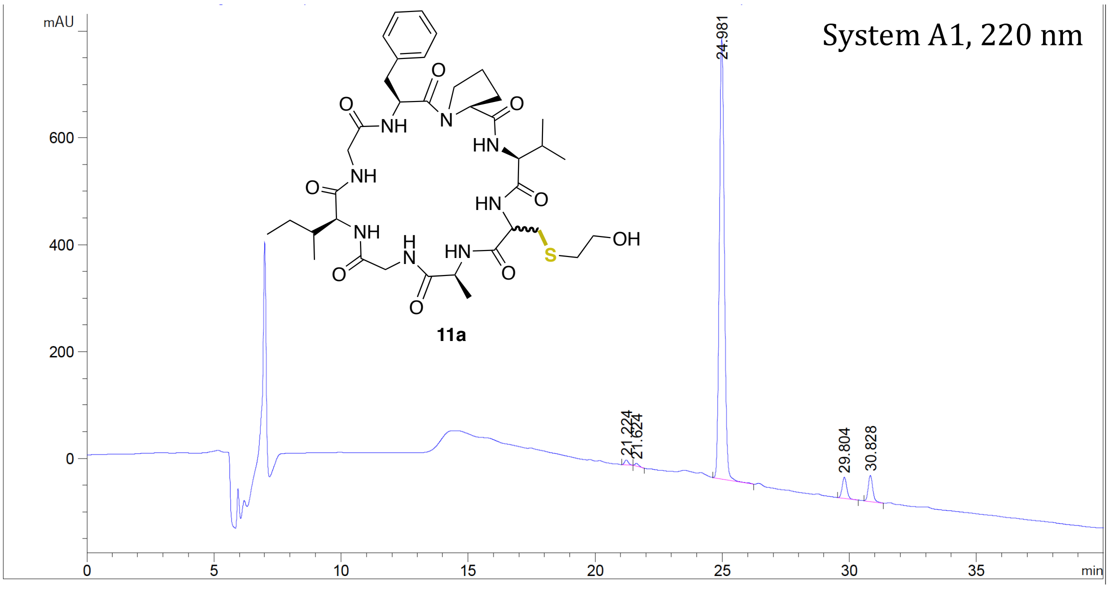
System A1, 220 nm
mAU
600
400
200
0
0
5
10
15
20
25
30
35
min
24.981
21.224
21.624
29.804
30.828
11a

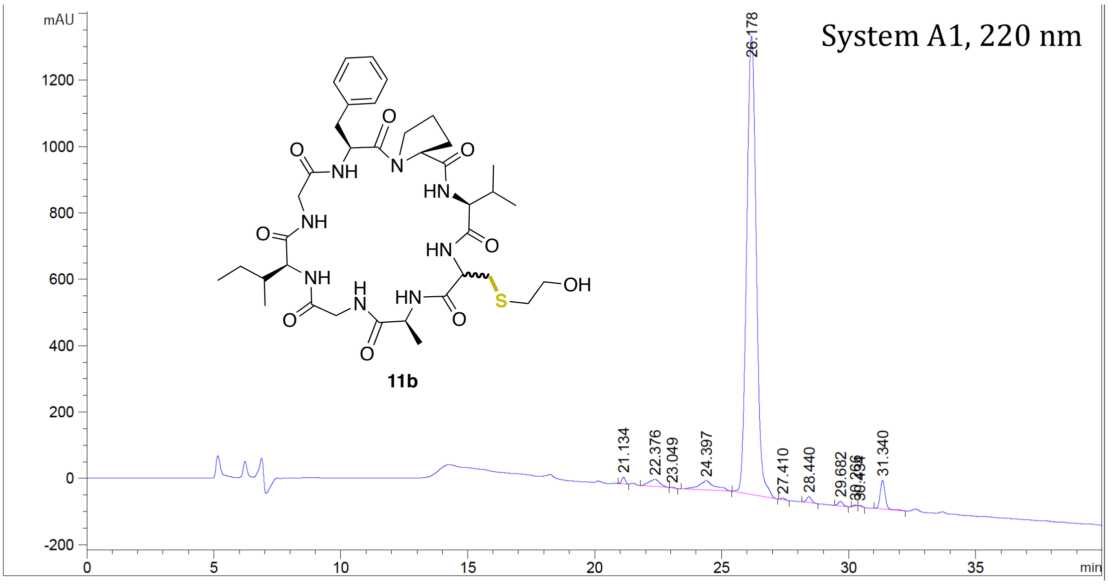
System A1, 220 nm
mAU
1200
1000
800
600
400
200
0
-200
0
5
10
15
20
25
30
35
min
26.178
21.134
22.376
23.049
24.397
27.410
28.440
29.682
31.340
11b

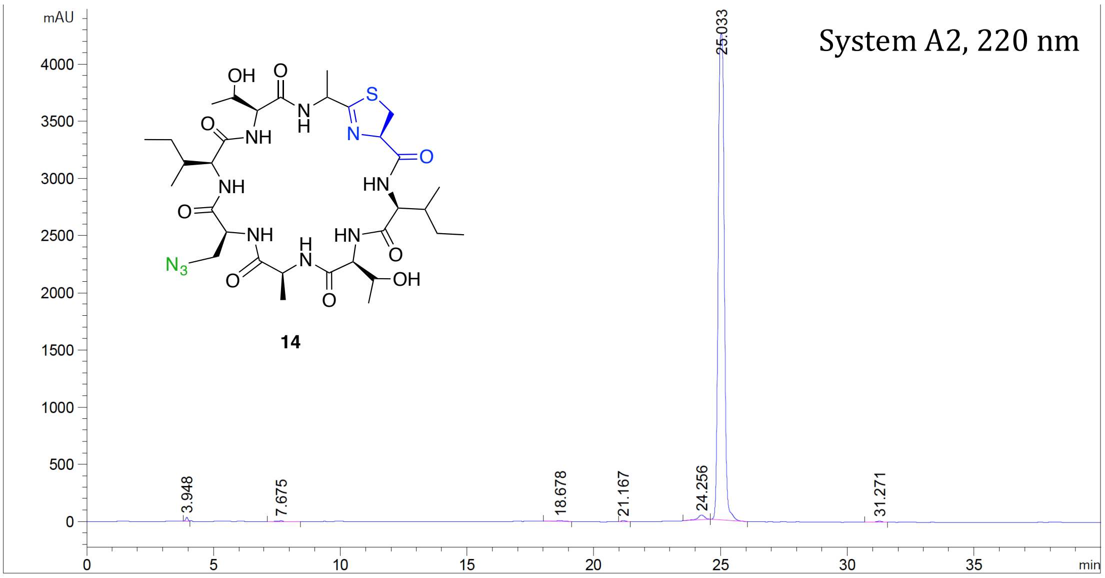
System A2, 220 nm
mAU
4000
3500
3000
2500
2000
1500
1000
500
0
0
5
10
15
20
25
30
35
min
25.033
3.948
7.675
18.678
21.167
24.256
31.271
14

VII. References

(1) Fulmer, G. R.; Miller, A. J. M.; Sherden, N. H.; Gottlieb, H. E.; Nudelman, A.; Stoltz, B. M.; Bercaw, J. E.; Goldberg, K. I. *Organometallics* **2010**, *29*, 2176.

(2) Koehnke, J.; Bent, A.; Houssen, W. E.; Zollman, D.; Morawitz, F.; Shirran, S.; Vendome, J.; Nneoyiegbe, A. F.; Trembleau, L.; Botting, C. H.; Smith, M. C. M.; Jaspars, M.; Naismith, J. H. *Nat. Struct. Mol. Biol.* **2012**, *19*, 767.

(3) Koehnke, J.; Mann, G.; Bent, A. F.; Ludewig, H.; Shirran, S.; Botting, C.; Lebl, T.; Houssen, W. E.; Jaspars, M.; Naismith, J. H. *Nat. Chem. Biol.* **2015**, DOI: 10.1038/nchembio.1841.

(4) Lau, Y. H.; Spring, D. R. *Synlett* **2011**, *13*, 1917.

(5) Final peptide purchased from Peptide Protein Research Ltd.

(6) Burrage, S. A.; Raynham, T.; Bradley, M. *Tetrahedron Letters* **1998**, *39*, 2831.
